# Experimental Murine Models for Colorectal Cancer Research

**DOI:** 10.3390/cancers15092570

**Published:** 2023-04-30

**Authors:** Íris Neto, João Rocha, Maria Manuela Gaspar, Catarina P. Reis

**Affiliations:** 1 Research Institute for Medicines (iMed.ULisboa), Faculdade de Farmácia, Universidade de Lisboa, Av. Prof. Gama Pinto, 1649-003 Lisboa, Portugal; irisneto@campus.ul.pt (Í.N.); jrocha@ff.ulisboa.pt (J.R.); 2 Instituto de Biofísica e Engenharia Biomédica (IBEB), Faculdade de Ciências, Universidade de Lisboa, Campo Grande, 1749-016 Lisboa, Portugal

**Keywords:** murine model, colorectal cancer, animal model, patient-derived xenografts, carcinogen-induced models, genetically engineered mouse models, metastatic model

## Abstract

**Simple Summary:**

In its early stages, colorectal cancer (CRC) is a localized tumor, but when it metastasizes, it has dramatic consequences. Murine models in CRC research are important tools for advancing the knowledge in diagnostic and treatment of this pathology. The present review aims to provide a variety of murine models in CRC research describing their particular advantages and drawbacks.

**Abstract:**

Colorectal cancer (CRC) is the third most prevalent malignancy worldwide and in both sexes. Numerous animal models for CRC have been established to study its biology, namely carcinogen-induced models (CIMs) and genetically engineered mouse models (GEMMs). CIMs are valuable for assessing colitis-related carcinogenesis and studying chemoprevention. On the other hand, CRC GEMMs have proven to be useful for evaluating the tumor microenvironment and systemic immune responses, which have contributed to the discovery of novel therapeutic approaches. Although metastatic disease can be induced by orthotopic injection of CRC cell lines, the resulting models are not representative of the full genetic diversity of the disease due to the limited number of cell lines suitable for this purpose. On the other hand, patient-derived xenografts (PDX) are the most reliable for preclinical drug development due to their ability to retain pathological and molecular characteristics. In this review, the authors discuss the various murine CRC models with a focus on their clinical relevance, benefits, and drawbacks. From all models discussed, murine CRC models will continue to be an important tool in advancing our understanding and treatment of this disease, but additional research is required to find a model that can correctly reflect the pathophysiology of CRC.

## 1. Introduction

Colorectal cancer (CRC) is the third most common malignancy and the second most deadly cancer worldwide. It is estimated that the number of cases in 2020 has reached c. 1.9 million with some 0.9 million deaths worldwide [1]. If there is no advancement in early detection and efficient therapies for late-stage CRC, this significant public health burden is anticipated to grow [2]. 

The etiology of CRC is complex and multifactorial. The disease is influenced by various genetic, environmental, and lifestyle factors that can increase an individual’s risk of developing the disease. The early diagnosis, detection and therapy of patients with CRC are vital (Table S1) [3,4,5,6,7,8,9,10,11,12,13,14,15,16,17,18,19,20,21,22]. Although extensive research has been performed in the last decade about CRC, important issues still need to be solved, such as early diagnosis of micro-metastases and chemotherapy resistance. In this sense, preclinical animal models are indispensable tools to answer these issues.

**Figure 1:**
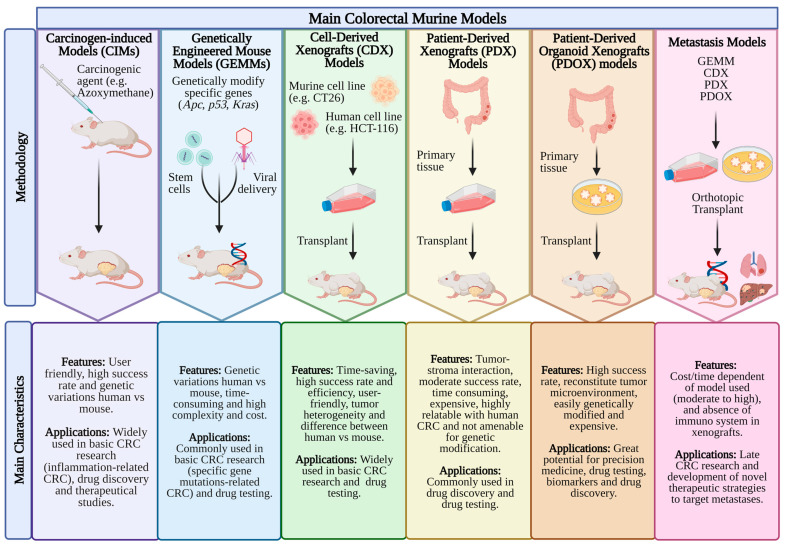
The frequently used mouse models in CRC research. The main features and applications of mouse models, including CIMs (carcinogen-induced models), GEMMs (genetically engineered mouse models), CDX (cell line-derived xenograft), PDX (patient-derived xenograft), PDOX (patient-derived organoid xenograft) and metastases models are summarized. Created with BioRender.com, accessed on 28 April 2023.

Animal models have become the foundation of CRC basic research, enabling the study of the disease’s pathogenesis and validating new therapies (Figure 1). The murine models show a resource with enormous promise since they allow researchers to simultaneously observe and control a complex disease such as CRC. The advantages of murine models include their low cost, manageability, short gestation period, anatomical resemblance to humans and ease of genetic manipulation. For research on carcinogenesis, quick tumor development, the ability to analyze the adenoma–carcinoma sequence, the use of transgenic, knock-out and knock-in animals are additional benefits [23,24,25]. Various murine models have been reported, but each one has limitations [26]. Additionally, there is animal-to-animal variation in the development of CRC in various murine models. Despite this, animal models have developed into a crucial tool for better understanding the impact of genetic changes on the disease process [27].

This review provides an overview of the most used CRC murine models, describing their particular benefits and drawbacks.

## 2. Materials and Methods

An extensive evaluation of the literature on murine models of colon carcinogenesis was conducted by scanning the PubMed database of the National Library of Medicine. MeSH phrases such as “colorectal cancer”, “animal model”, “chemoprevention”, “colon-carcinogenesis”, “min-mice”, “colorectal cancer”, “xenograft”, “heterotopic model”, “orthotopic model”, “metastatic model”, “patient-derived tumor”, “genetically engineered murine models”, and “transplant murine model” were used in the MEDLINE search. By using the aforementioned search terms, 9063 papers were initially identified from 1940 until the present. All of the 650 pertinent publications were carefully examined and studied and 273 of the manuscripts dealing with the murine models for CRC were included in this study. To help the reader, a full list of abbreviations used is presented in Abbreviations.

## 3. Carcinogen-Induced Models (CIMs)

Carcinogen-induced models (CIMs) have been used to study CRC for many years. These models involve the administration of a known carcinogen which results in a tumor [28]. CIMs have been used to elucidate the molecular pathways involved in CRC and to identify potential targets for the treatment [28]. Examples of carcinogenic compounds include: (1) methylazoxymethanol (MAM) azoxymethane (AOM), 1,2-dimethylhydrazine (DMH), (2) heterocyclic amines (HCAs) such as 2-amino-3-methylimidazo[4,5-*f*]quinoline (IQ) and 2-amino-1-methyl-6-phenylimidazo [4,5-b] pyridine (PhIP), (3) aromatic amines such as 3,2-dimethyl-4-aminobiphenyl (DMAB) and (4) alkylating substances such as N-methyl-N-nitro-N-nitrosoguanidine (MNNG) and methylnitrosourea (MNU).

The administration of these carcinogens is possible via ad libitum feeding, oral gavage (o.g.), intraperitoneal (i.p.), subcutaneous (s.c.) or intramuscular (i.m.), injections or intrarectal (i.r.). These chemical carcinogens can be administered alone or in combination. Some carcinogens require biotransformation to cause cancer, while others do not. In contrast to direct agents, indirect carcinogens are delivered in an inactive state and acquire carcinogenic activity only after being biotransformed into their active form in the liver [29]. Intestinal mutations are the key to the success of the CIMs. Tumors from these animals randomly develop genetic and pathological similarities to human CRC [30]. Typically, this model takes a considerable amount of time to establish the type of cancer, because these tumors progress slowly from normal cells to adenocarcinoma/carcinoma [28]. In addition, the incidence of CRC development is influenced by the rodents’ gender, age, and genetic background. Additionally, the intestinal flora, nutrition and immunological condition of rodents can interfere with the metabolism of carcinogenic chemicals, consequently affecting their effective local concentration. Relevant research has been summarized in Table 1.

The general characteristics of the murine models and the mechanisms by which these carcinogens cause CRC are addressed in the following section.

### 3.1. 1,2-Dimethylhydrazine (DMH)

DMH and azoxymethane (AOM), a metabolite of DMH, are procarcinogens that must undergo metabolic activation to produce DNA-reactive by-products. The methylation of guanine at position N-7 in DNA is the starting point for the mutagenic activity of the alkylating agents DMH and AOM. By providing a proton, the alkylated guanine is coupled with thymidine rather than cytosine, changing the bases [31]. DNA mutations result from further replication, mismatching of guanine to thymine and cytosine to adenine, and other events. Different metabolic enzymes, such as xenobiotic-metabolizing enzymes, process these procarcinogenic chemicals’ N-oxidation and N-hydroxylation steps, resulting in the creation of the final carcinogen, MAM [31], a reactive metabolite of DMH and AOM [29].

There are different routes that DMH can be administered such as o.g., s.c., i.p., and i.r. [29]. In Table 1 several examples using DMH are listed, including the animal species, dose injected, latency time and main characteristics of developed tumor.

The formation of tumors in the colon can be induced by DMH at a wide range of dosing levels from 2 mg to 200 mg/kg b.w. (a single injection to 30 injections) and with a latency period from 8 h to a maximum of 78 weeks (critical for the development of a tumor in the colon) (Table 1). Recently, routes of administrations and dosages were standardized (15 and 20 mg/kg b.w.) depending on the experimental study [32].

Despite the fact that most experimental CRC research has been conducted in murine animals, the high frequency of tumors in the lower part of the colon and the histopathological evidence of multiple adenomas and subsequent progression of adenocarcinoma validate the importance of DMH-induced models in the pathogenesis of CRC [31].

Although DMH-induced colon tumors in rodents are comparable to human colon tumors, this model has some drawbacks, such as the requirement for numerous DMH injections to cause tumors [27], the presence of a latency phase lasting at least six months and the absence of hepatic metastases up until this point [29] with non-transgenic rodents.

#### DMH and Colitis-Associated CRC (CAC) Models

CAC is a consequence of inflammatory bowel disease (IBD) with a poor prognosis since it is frequently identified in advanced stages with local development or metastases. CAC has different molecular processes than polyp-induced sporadic CRC (sCRC), which is more prevalent [33]. Although a full understanding of IBD pathogenesis is unclear, the predominant pathological finding is characterized by persistent inflammatory processes at the local site [34]. Previous research has demonstrated that local immunological processes during chronic inflammation are distinct in ulcerative colitis (UC) and Crohn’s disease (CD) [34,35]. CD is characterized by the presence of activated T helper type 1 (Th1) cells in the intestine, as well as high expression of interferon gamma (IFN-γ) and tumor necrosis factor alpha (TNF-α) [34,35]. On the other hand, enhanced expression of cytokines produced from T helper type 2 (Th2) cells is frequently observed in UC [34,35].

The most common chemical agents to generate CAC models are dextran sulphate sodium (DSS), which promotes a Th2 response that is similar to UC, and 2,4,6-rinitrobenzenesulfonic acid (TNBS), which promotes a Th1 response that is similar to CD [36,37]. Some examples are listed in Table 1. For instance, male Balb/c mice received a single dose i.p. of DMH (20 mg/kg b.w.), and one week after, DSS (3%) was given in drinking water for 1 week followed by normal drinking water for next 2 weeks (three alternate cycles of DSS), and at week 20, mice were euthanized [38]. In this study, upregulation of all the signaling events in DMH/DSS mice indicates the development of an aggressive and invasive carcinoma, mainly driven by nuclear factor kappa B (NF-κB) and also the loss of goblet cells that result in decreased production of goblet cell specific mucin such as MUC2, which are associated with poor prognosis in CRC. The reduction in both acidic and neutral mucins in DMH/DSS-treated mice suggested the advanced stage of tumor progression with carcinogen treatment [38].

In a DMH/TNBS model, male Wistar rats received twice every week four doses of DMH (40 mg/kg b.w) and two weeks after receiving DMH, 10 mg of TNBS mixed in 0.25 mL of 50% ethanol (*v*/*v*) was administered i.r. in the middle colon to generate acute inflammation and expedite the carcinogenesis process [39]. By week 25, the rats were euthanized and showed tumors with low grade dysplasia (60%) and high grade dysplasia (40%), a tumor multiplicity of 3.50 ± 1.72, and aberrant crypt foci (ACF) [39].

### 3.2. Azoxymethane (AOM)

Given its benefits over the original compound, AOM has been utilized more frequently than DMH in the induction of CRC [40]. It is suggested that AOM induces colon carcinogenesis more effectively than other carcinogens due to its greater stability, with enhanced potency being one of its advantages [36]. Similar to DMH, AOM is a procarcinogen that is activated in the liver by N-oxidation via cytochrome P450 2E1, producing proinflammatory metabolites such as methylazoxymethanol and methyl-diazoxide; however, it seems that AOM acts like a more efficient carcinogen [41]. A drawback of the AOM approach includes the fact that this procarcinogen is more expensive than DMH, highly toxic, and has stringent shipping requirements, despite its widespread use [33].

AOM promotes CRC in rodents when injected i.r., s.c. or i.p. as described throughout the literature; however, the preferred form of administration of this procarcinogen is i.p. The distribution of small intestine and colon tumors (mostly in the distal colon) is comparable to that reported in the human colon [42]. AOM-induced tumors share the same histological and histochemical characteristics as human cancers, being classed as adenomas and adenocarcinomas [43].

Rosenberg et al. found that mice with different genetic backgrounds have distinct sensitivity to AOM, with A/J and SRJ/R mice having great sensitivity (8 weeks after AOM treatment), while C57B/L6 and Balb/c mice have moderate sensitivity [27]. The tumor morphology was similar among these two mouse strains, while metastases or invasion was not observed even in the mouse strain with high AOM sensitivity. Histopathological progression varies among these two mouse strains, with tumor crypts composed of closely packed cells with indistinct cell borders and infiltration of neoplastic cells into the muscular wall of the distal colon. This model is suitable for studying early-stage but not late-stage or metastatic CRC [43].

As a result of their high similarity to human CRC, AOM-induced CRC tumors and carcinogenesis in murine animal models are widely used to evaluate novel chemopreventive and therapeutic strategies and to provide new insights into the risk factors and pathophysiological mechanisms of human CRC [44].

#### AOM and Colitis-Associated CRC (CAC) Models

The combination of AOM and DSS is a reliable method for inducing CRC in mice, replicating the pathogenesis of CAC, and is thus a highly reproducible acute or chronic model of intestinal colonic inflammation [36,45]. The standard protocol for DSS-induced colitis in rodents involves addition of DSS to drinking water at a concentration of 2–10% (3–5 cycles). This model is also helpful for gaining insight into the innate immune mechanisms of UC [36]. Table 1 also lists some examples. A disadvantage of this model is that it has a relatively low propensity of developing metastases [46]. Furthermore, the AOM/DSS model’s immunological characteristics may not reflect those of typical human CRC cases, making it inappropriate for preclinical research [28].

Although TNBS model is a readily inducible, fast, robust and highly reproducible CAC model [36], it is not frequently used in association with AOM. Nevertheless, Xiao et al. used this model with 8-week-old C57BL/6 mice. The authors used AOM (10 mg/kg b.w., i.p.) followed by 2.5 mg i.r. of TNBS (150 µL with 50% EtOH) [47]. The results showed that the mice displayed extensive inflammatory, dysplasia, or carcinoma lesions all over entire mucosa with numerous ulcers and edema of submucosal and muscle layers. In addition, an overexpression of proinflammatory cytokines TNF-α and IL-12 were also observed.

Variations in susceptibility to TNBS-induced colitis among mouse strains demand optimization of the concentration of TNBS. Individual adjustment of inflammatory agent doses is necessary. Although the experimental protocols are straightforward in concept, individual variances in the intestinal microbiota between animal facilities and the genetic variety of mice strains necessitate pilot studies to adjust the dosages of TNBS and DSS [36,37]. Additionally, substantial variation between batches of these chemicals is possible.

### 3.3. Heterocyclic Amines (HCAs)

In rodents, HCAs, such as IQ and PhIP, are mutagens that are created when meat and fish are broiler-cooked [41,48]. Creatinine, amino acids and carbohydrates found in meat and fish are the precursors of IQ-type HCAs. For IQ to transform into its ultimate carcinogen, liver microsome metabolic activity is required [49]. Cytochrome P450s in the liver convert an amino group to a hydroxyamino group, which activates HCAs, and these are further triggered, resulting in DNA adducts that cause cancer [50]. IQ and PhIP have attracted a significant amount of interest due to their multitarget organ specificity, as they induce cancer in the stomach, colon, mammary gland and prostate of rodents [27,51].

Dietary treatment of IQ and PhIP to rodents for 52 weeks resulted in a low incidence of tumors (5–28%); however, feeding rodents PhIP for 104 weeks was associated with a high incidence of colon tumors (43–55%) and significant toxicity [52]. Malignancy type and onset time appear to be highly dependent on the experimental model; however, in all models, spontaneous tumor induction by PhIP alone requires lengthy periods of time, typically between 52–82 weeks [53]. Nevertheless, when PhIP is paired with DSS, colon adenomas and adenocarcinomas arise in rodents, and the duration of tumor initiation is decreased to 6–24 weeks, depending on the animal strain [53]. Additionally, to stimulate tumor growth, PhIP can be used alone or in conjunction with AOM and DMH.

This model is utilized more frequently in chemoprevention research since this carcinogen may be ingested by humans [53]. As early as 10 weeks after PhIP/DSS administration, histopathologic and biochemical analyses revealed tubular adenocarcinomas with substantial overexpression of critical proteins in the Wnt signaling pathway (e.g., *β*-catenin), cell regulation (e.g., *c-Myc* and cyclin D1), and inflammation (e.g., iNOS and COX-2), thus providing one advantage of this model when studying the overexpression of the proteins above mentioned [54,55].

### 3.4. Aromatic Amines

The first intestinal cancer was chemically induced in mice fed with the polyaromatic hydrocarbon 3,2-dimethyl-4-aminobiphenyl (DMAB) or methylcholanthrene [56,57]. The DMAB model has two main disadvantages: a) multiple injections are generally required; and b) low specificity since tumors may be induced in other tissues such as adenocarcinoma of mammary glands, urothelial carcinoma of the bladder, sarcoma, lymphoma, among others [41,52]. In rodent models, DMAB is less efficient than the series of compounds formed from DMH or AOM on a molar equivalent basis [27].

Several studies were made with DMAB and some of them are listed in Table 1. The murine model that stands out was performed by Reddy et al. with male F344 rats who received weekly s.c. injections (50 mg/kg b.w.) for 20 weeks [58]. The rats were autopsied after the last injection of DMAB and the histopathological findings revealed adenocarcinomas. This study found that diets containing wheat bran and citrus fiber reduce the risk for DMAB-induced intestinal cancer and that the protection against CRC depends on the type of fiber. DMAB reacts with DNA through the formation of DNA adducts via N-hydroxylation, O-acetylation, and hydrolysis by cytochrome P450 [59].

To cause cancer, heterocyclic and aromatic amines undergo metabolic activation by the enzymes NAT1 and NAT2. A high frequency of NAT1 and NAT2 polymorphisms makes humans prone to cancer when exposed to aromatic and heterocyclic carcinogens [60]. F-344 and WKY rats received a s.c. injection of DMAB (50 or 100 mg/kg b.w. in peanut oil) in weeks 1 and 2 and were euthanized at week 10, demonstrating a higher frequency of ACF in colonic tissue of rapid acetylator (NAT2) genotype, thus showing it to be a risk factor in aromatic amine-induced colon carcinogenesis [61].

In another study, male F344 rats (5-weeks old) received a s.c. injection of DMAB (100 mg/kg b.w. in peanut oil), and 48 h after, the rats were euthanized and DMAB-derived adducts were analyzed in colon and liver [59]. This study investigated the chemoprotective effect of celecoxib on levels of DMAB-derived adducts in the target organ colon and the non-target liver. DMAB shares structural similarities with mutagens found in well-done meat and has been demonstrated to be affected by dietary fat and fiber in F344 rats. DNA adduct development is vital, but additional elements such as cell proliferation are required for tissue susceptibility to tumor development. Comparable quantities of adducts were found in the liver and colon; however, in this model, cancer only occurs in the colon, and celecoxib showed a related dose-dependent decrease in DMAB-derived DNA adducts.

### 3.5. Alkylating Substances

N-methyl-N-nitro-N-nitrosoguanidine (MNNG) and methylnitrosourea (MNU) are direct alkylating substances, i.e., they do not need metabolic activation. MNU or MNNG injected intrarectally has been shown to cause CRC in rat studies [62,63]. For instance, i.r. treatment with MNNG (1–3 mg/week b.w.) for 20 weeks caused CRC at the injection site in male F344 rats (57% adenomas and 43% adenocarcinomas) [29,41,49]. MNU, despite developing CRC when administered i.r., may also induce thymic lymphoma and lung malignancies, which can be fatal [64]. It has been demonstrated that the development of DNA adducts and abnormal crypt foci are the results of i.r. administration of MNU [65].

CRC induced by MNU or MNNG contains *Kras* (5–30%) and *Apc* (6%) mutations; nevertheless, the complete molecular profile of mutations caused by these carcinogens is still unknown [66,67,68]. Since i.r. administration of MNNG and MNU selectively produces tumors in the distal colon and rectum, these models have been widely utilized to test the therapeutic effects of various drugs for CRC management [29].

The primary drawback of the alkylnitrosamines models is the difficulty in administering a precise dose of the carcinogens *per* rectum [27,29,41]. Additionally, the animals must remain inverted for one minute following administration to prevent the carcinogens from returning to the anus [27,29].

Furthermore, to clarify the carcinogenic factors (such as the type of carcinogen and the duration of exposure) that determine whether CRC develops from an adenoma, occurs de novo, or in the absence of an adenoma, Endo et al. performed a comparative histopathological study of DMH (30 mg/kg b.w., i.p., once a week) and MNNG (10 mg/kg b.w., i.r., 3 times a week) for 3 or 15 weeks in male F344 rats [66]. In the 3-week group, low grade dysplasia coexisted with 71% of DMH-induced carcinomas and 82% of MNNG-induced carcinomas, while this was observed in only 10% of DMH-induced and 27% of MNNG-induced carcinomas in the 15-week groups, and the low-grade dysplasia predominated from the initial period of tumor occurrence. The study also investigated whether or not mutations in the *Kras* and *Apc* genes were linked to these carcinogenesis patterns. No *Kras* mutations were found in tumors that had been exposed for 3 weeks. Nevertheless, this mutation was found in 57% of DMH-induced tumors and 13% of MNNG-induced tumors in the 15-week group. Only 6% of tumors had mutations in an area of *Apc* that is similar to the human mutation cluster area. These findings provide further evidence that the patterns of carcinogenesis in the rat colon are time-dependent and that *Kras* mutations contribute partly to a subset of the patterns. Table 1 summarizes some murine models induced by chemical carcinogens.

**Table 1:** CRC carcinogen-induced models (CIMs).

Carcinogen	Animal Strain and Gender	Dose/Route	Latency Period	Tumor Characteristics	Ref.
DMH	Swiss albino mice	10 mg/kg b.w./wk, s.c.	17 wks	Hyperplasia with irregular-shaped mucosa, distorted crypts and laminar cellular infiltration (*CD31* and *Vegf*)	[69]
	Female Wistar rats	20 mg/kg b.w./wk, s.c.	30 wks	Adenocarcinoma; ACF, MDF and disintegration of goblet cells (NF-κB, iNOS, *β*-catenin, PCNA, COX-2, *Bax*, cleavedPARP, *Bcl-2*, *Apc*)	[70]
	Male Wistar albino rats	20 mg/kg b.w./wk, s.c. or i.r.	15 wks	Tumor cells indicative of anaplasia, dysplasia and hyperchromasia in the lumen (*Krt20*, SOD, CAT, *B*ax *Bcl-2*, caspase-3, cytochrome C, iNOS, TNF-α/β, IL-1β and COX-2)	[71]
	Male Balb/C mice	20 mg/kg, b.w./wk, s.c.	30 wks	Adenomas and adenocarcinomas	[72]
	Male Wistar rats	40 mg/kg b.w./2 times a wk, s.c.	20 wks	Signet-ring cell carcinoma (*p53*, *PI3K*-*Akt*, IKK/NF-κB, MAPK and intrinsic apoptotic signaling pathways bioinformatics analysis)	[73]
	Female CD1 Swiss albino mice	20 mg/kg b.w./wk, s.c.	20 wks	Tubular adenoma, dysplasia and anal squamous cell carcinoma (inflammation markers (IL-17, IL-10, TGF-β))	[74]
	Male albino Balb/c mice	20 mg/kg, b.w./wk, i.p.	24 wks	Adenoma and adenocarcinoma (Wnt pathway, COX-2, iNOS)	[75]
	Male Fisher rats	35 mg/Kg, b.w., o.g.	78 wks	Adenocarcinoma	[76]
	C57B1/6J and mice	10, 20 and 50 mg/kg b.w., i.p. or o.g.	24 h	Nuclear aberration (NA)	[77]
	Male Wistar rats	40 mg/kg b.w., i.p./wk	10 wks	Wnt signalling pathway (e.g., *β*-catenin and *p53*), cell regulation (e.g., *c-Myc* and cyclin D1), inflammation (e.g., IL-6, ROS and COX-2) and alterations of bacterial enzymes (e.g., *β*-glucuronidase and *β*-glucosidase)	[78]
DMH/TNBS	Male Wistar rats	DMH (40 mg/kg b.w./ 2 times a wk, s.c., 2 wks); TNBS (10 mg in 0.25 mL of 50% ethanol (*v*/*v*), i.r.)	25 wks	Adenocarcinoma (*Ki-67*, *β*-catenin, *Cx43*, *Msh6*, *Ppara*, *Akt3*, *Dlc1* and *Vegfd*)	[39]
DMH/DSS	Male Wistar rats	DMH (30 mg/kg b.w./single dose, i.p.; 1 week after 2% (*w*/*v*); DSS in drinking water for 7 days	18 wks	Adenoma (apoptosis-associated *p53*/*Bcl-2*/*Bax* signaling)	[79,80]
	Male BALB/c mice	DMH (20 mg/kg b.w./wk, i.p., wks 0, 3 and 6); DSS (3% *w*/*v*, 3 cycles) for 7 days (2 wks gaps)	10 wks	Aberrant crypts, loss of goblet cells and increased cell infiltration (SOD, Nrf2, NF-κB, Caspase-1, STAT-3 and IL-6 expression)	[81]
	Male F344 rats	DMH (40 mg/kg b.w./3 times a wk, i.p.); DSS (2% in drinking water) for 1 wk	10 wks	Preneoplastic ACF and MDF (SOD, *Bcl-2*, *p53*, *Bax* and caspase-3 expression)	[82]
AOM	Female A/J mice	10 mg/kg b.w./wk, s.c.	16 wks	(*Hif-1a*, *Aldoa*, *Pgk1*, *Raptor, Dek* and *Vegf* expression)	[83]
	Male C57BL/6 mice	10 mg/kg b.w./single dose, i.p.	9 wks	Adenoma (*Ki-67* and *PCNA* protein expression; IFN-γ, IL-6, TNF-α, Th1 and Th17)	[84]
	Balb/c mice	15 mg/kg b.w./single dose, i.p.	8–9 wks	Adenoma and adenocarcinoma (pro-apoptotic (cytochrome C, *DR4*, *DR5*, TNFRSF1A, *Bax* and *BAD*) and anti-apoptotic proteins (*Hsp70*, *Hsp32*, and *XIAP*))	[85]
	Male Sprague Dawley rats	7 mg/kg b.w./wk, s.c.	8 wks	ACF dysplastic and hyperplastic	[86]
	A/J mice	8 mg/kg b.w./wk, i.p.	12 wks	Adenoma–carcinoma sequence	[87]
	Male Balb/c mice	10 mg/kg b.w./wk, i.p.	25 wks	Adenocarcinoma (*PI3K/Akt/mTOR* pathway)	[88]
	Male Wistar rats	15 mg/kg b.w./wk, s.c..	37 wks	Adenoma and adenocarcinoma (metastases-associated in colon cancer 1 (MACC1))	[89]
	C57BL/6J and KKAy	(10 mg/kg b.w./wk, i.p.	6 wks	Polyps, adenocarcinomas and ACF	[90]
	Male Wistar rats	15 mg/kg b.w./wk, s.c.	2 wks	Numerous large ACF with hyperplastic and dysplastic features, precancerous mucin-depleted foci (MDF) and multiple tubular adenomas	[44]
	A/J mice	10 mg/kg b.w./wk, i.p.	6 wks	Multiple tubular adenoma (overexpression of *Hif-1a*, *Aldoa*, *Pgk1* and *Vegf* genes)	[83]
AOM/DSS	Male C57BL/6 mice	AOM (12.5 mg/kg b.w./single dose, i.p.); DSS (2.5% in drinking water) for 5 days at wks 2, 6 and 9	12 wks	Adenoma (inflammation markers (IL-1β, IL-8, IL-10, TNF-α), claudin-1, *β*-actin, NF-κB and p38 MAPK pathways)	[91]
	Male C57BL/6 mice	AOM (10 mg/kg b.w./single dose, i.p.); DSS (2.5% in drinking water) for 1 wk at wks 2, 5 and 6	10 wks	Adenoma (Inflammation markers (IL-6, IL-1β, COX-2 and TNF-α), cell-proliferation marker *Ki67*, tight junction proteins (ZO-1 and occludin) and Wnt/*β*-catenin pathway)	[92]
	Female Balb/C and C57/Bl6 mice	AOM (12.5 mg/kg b.w./single dose, i.p.); DSS (1, 2, or 3% (*w*/*v*) in drinking water) for 5 days at wks 2, 5 and 8	12 wks	Carcinomas (3% DSS) (cell-proliferation marker *Ki67*)	[93]
	Female FVB/NJ mice	AOM (10 mg/kg b.w./single dose, i.p.); DSS (3% in drinking water, 2 cycles) for 7 days	8 wks	Adenoma (cell-proliferation marker *Ki67*; inflammation markers (IL-6, IL-10, IL-22, IL-1β, IL-17α and TNF-α)	[94]
	Male F344 rats	AOM (15 mg/kg b.w./1 time a wk, i.p., 3 wks); DSS (3% in drinking water, 2 cycles) for 7 days	21 wks	Adenocarcinoma (microbiome-community phylogenetic analysis)	[95]
	Male Wistar rats	AOM (10 mg/kg b.w./1 time a wk, s.c., 2 wks); DSS (4% in drinking water, 2 cycles) for 7 days	10 wks	Adenoma and adenocarcinoma (inflammation markers (IL-6, IL-10, COX-2, NF-κB) and Wnt/*β*-catenin signaling pathway)	[96]
	*Lgr5* ^eGFP-IRES-CreERT2^ mice	AOM (10 mg/kg b.w., i.p.); DSS (2% in drinking water; 3 cycles) for 5 days	11 wks	Adenoma (*Ly6a* (Sca-1), *Tacstd2* (Trop2) and Sox9 gene expression	[97]
AOM/TNBS	C57BL/6 mice	AOM (10 mg/kg b.w./single dose, i.p.); 2.5 mg of TNBS (150 µL 50% EtOH) i.r.	NR	Extensive inflammatory, dysplasia or carcinoma lesions all over entire mucosa with numerous ulcers (TNF-α, IFN-γ, IL-1β and anti-inflammatory cytokines IL-10 and IL-12)	[47]
	IFN-γ^−/−^ and IL-4^−/−^ mice	AOM (10 mg/kg b.w./1 time a wk, i.p., 3–6 wks); TNBS (2% PBS:ethanol (1:1), i.r., 3–10 wks)	33 wks	Adenocarcinomas (*p53*, *β*-catenin, Th1 and Th2)	[35]
PhIP	hCYP1A mice	PhIP (0.01–200 mg/kg b.w., o.g. DSS (1.5% (*w*/*v*) in drinking water for 5 days)	8 wks	Adenoma (*p53* signaling network and regulatory pathways)	[98]
	hCYP1A mice	PhIP (100 mg/kg b.w./2 doses, i.g. with 3 days apart); DSS (1.5% (*w*/*v*) in drinking water for 4 days)	10 wks	Adenocarcinoma (oxidative and nitrosative stress markers (8-oxo-dG and nitrotyrosine) and inflammation markers (NF-κB and p-STAT3)	[99]
MNNG	Female C57BL6 mice	100 mg/kg b.w., i.r.	12 wks	Adenoma–carcinoma sequence (endoscopic evaluation)	[25]
	Male BALB/c mice	4 successive dosages (5 mg/mL; i.r. deposits of 100 µL, twice a wk for 2 wks	10 wks	Adenocarcinoma (*PCNA*, COX-2, IL-12, IL-10, TNF-α and INF-γ)	[100]
	Male C57/BL6 mice				
	Male IL-10^−/−^ mice				
	Female C57BL/6 mice	4 successive dosages (5 mg/mL; i.r. deposits of 100 µL, twice a wk for 2 wks	8 wks	Adenoma and adenocarcinoma (*PCNA*, *Ki67*, *c-Myc*, *Vegf*, *CD133*, *CD34* and *CD31*)	[101]
MNU	Male albino Wistar rats	1.2% in 1.9% citric acid, i.r.	12 wks	Adenoma (MLH-1 and SOD)	[102]
	Male Wistar rats	8 mg/kg b.w., 3 times a wk, 4 wks, i.r.	25 wks	Adenocarcinoma and signet ring cell carcinoma (*Kras*, *Ki67* and caspase-3 expression; IFN-γ, IL-1β, IL-8, TGF-β, TNF-α and IL-6; Wnt-*Apc*-*β*-catenin pathway)	[103]
	Female Sprague Dawley rats	10 mg/kg b.w., 3 times a wk, 4 wks, i.r.	NR	Adenoma (*PI3K/AKT*/*Bcl-2* pathway)	[104]
	Male Wistar rats	8 mg/kg b.w., 5 times a wk, 6 wks, i.r.	8 wks	FRZ-8, GAPDH, *Apc* gene expression, Wnt-*Apc*-*β*-catenin pathway	[105]
	Male Sprague-Dawley rats	8 mg/kg b.w., 3 times a wk, 5 wks, i.r.	16 and 24 wks	16 wks—Adenoma; 24 wks—Adenocarcinoma (Wnt/*β*-catenin and Notch pathways)	[106,107]
	Male F344/DuCrj rats	8 mg/kg b.w., 3 times a wk, 4 wks, i.r.	20 wks	ACF (*PCNA*)	[108]

DMH—1,2-dimethyl hydrazine; AOM—azoxymethane; DSS—dextran sulphate sodium; TNBS—2,4,6-trinitro benzene sulfonic acid; PhIP—2-amino-1-methyl-6-phenylimidazo [4,5-b] pyridine (PhIP); MNNG—N-methyl-N-nitro-N-nitrosoguanidine; MNU—methyl nitroso urea; b.w.—body weight; s.c.—subcutaneous; i.r.—intrarectal; i.p.—intraperitoneal injection; wk—week; wks—weeks; mos—months; h—hours; o.g.—oral gavage; i.g.—intragastric gavage; NR – Not reported.

## 4. Genetically Engineered Murine Models (GEMMs)

Numerous genes are involved in CRC including the tumor suppressors *Apc*, *DCC*, *p53* and *MCC*; the oncogenes *Kras*, *SRC,* and *C-myc*; the DNA repair genes *hMsh2*, *hMsh6*, *hMlh1*, *hPms1,* and *hPms2*; in addition to *CD44* genes and COX-2 [109].

Mutations in two or more of those genes are frequently associated with the malignant phenotype of CRC. Each of these genes plays a unique role in the formation of CRC tumors [109].There are many genetically engineered murine models (GEMMs) that are used in CRC research (Table 2). The most common model is the mouse with a mutated *Kras* gene, which is found in 86% of human CRC [110,111]. This model is used to study the development and progression of CRC, as well as the effectiveness of potential treatments. Other GEMMs that are used in CRC research include mice with mutations in the *Apc* gene, which is found in 90% of human CRC [112], and mice with mutations in the *p53* gene, which is found in 60% of human CRC [113].

All these models are used to study the role of these genes in the development and progression of CRC, as well as the effectiveness of potential treatments.

GEMMs are also frequently used to study CRC in addition to the CRC CIMs, TMMs, and metastatic models. 

The general characteristics of the APC mutant murine model and other transgenic animals for the investigation of CRC are covered in the following section.

### 4.1. Adenomatous Polyposis Mouse Models (APMM)

Carcinogenesis is a multi-step genetic process. It was shown that the mutation of the *Apc* gene is the first step in the carcinogenesis process of human CRC. The *Apc* gene controls a wide variety of cellular processes, including *β*-catenin levels, cytoskeleton organization, cell cycle regulation, apoptosis and adhesion [114]. Germline mutations in this gene are related to familial adenomatous polyposis (FAP) and CRC tumors [115].

Adenomatous Polyposis Mouse Models (APMM) are useful tools for the pre-clinical assessment of CRC therapies. These models involve mice with a genetic mutation causing them to develop multiple adenomas in the colon and rectum, similar to humans [43,116]. The tumors range from early neoplasia to advanced adenocarcinomas, allowing for realistic assessment of CRC treatments via targeted therapies, chemotherapy or combination therapies. These models allow a direct comparison between pre-clinical observations and clinical trial results by providing an in vivo platform which accurately replicates human tumor characteristics [117].

APMM have been particularly useful for understanding the molecular pathways and therapeutic targets involved in the development and progression of CRC carcinoma [43,118]. Several other genes such as tumor suppressors (*p53*, *Itf*, *Cables1*, *CpG-island* and *Tgfβ*), mismatch repair (MMR) (*Mlh1*, *Msh2*, *Msh6* and *Pms2*), multidrug resistance (*Mdr1*), autophagy-related (*Atg5*), trefoil factor family (*Tff2*), integrin gene (*Mac-1*), and others (*EphB*) are either directly involved in the growth of multiple intestinal polyps or CRC in the *Apc ^Min/+^* murine model, or indirectly affect this process [115,117].

*Apc ^Min/+^* model applications have progressed most in the areas of CRC-tumor prevention and treatment using primarily chemical and pharmaceutical strategies [115]. The histological analysis of *Apc* mutation-induced tumors of the colon revealed that they are benign adenomas, making this model appropriate for investigating the premalignant rather than malignant phases of CRC. Nevertheless, tumor malignancy increases, and latency time shortens when AOM or other carcinogenic compound is administered [117,119,120]. Despite the fact that additional *Apc*-targeting murine models have been developed (*Apc^∆716^*, *Apc^∆14^*, *Apc^1638N^*, among others), *Apc^Min/+^* continues to be the most widely employed transgenic murine model of CRC [43].

Cre-loxP is a site-specific recombinase technology used to carry out deletions, insertions, translocations, and inversions at specific sites in DNA. Cre-loxP-mediated murine models were developed to allow the tissue-specific and conditional knock-out of tumor suppressor genes or activation of oncogenes, respectively, overcoming the obstacles of frequent embryonic lethality caused by germline knock-outs of tumor suppressors [117]. Deleting *Apc* in Lgr5+ ISCs mice using Cre-LoxP results in the rapid development of intestinal adenomas. This fact suggests that Lgr5+ ISCs are the cells of origin for intestinal cancer.

Colon adenocarcinoma can develop in mice that carry a combination of *Apc^Min/+^* and *Smad*^−/−^ or *Apc^Δ716/+^* and *Smad4^+/^*^−^; however, *Apc^Min/+^* and *Apc^Δ716^* alone only induce the formation of adenomas and not invasive tumors [30,43]. Researchers are now able to regulate the timing and/or location of *Apc* deletions due to Cre-loxP technology. Cre recombinase deletions of *Apc* are restricted to the epithelial cells lining the gastrointestinal tract when expressed from tissue-specific promoters such as the *Fabpl-* and *Villin*-promoters [42,121]. To investigate the role of *p53* in colon tumor invasion, Cre-LoxP was used to create *Apc^fl/+^p53^fl/+^* and *Apc^fl/+^p53^R172H/+^* mice, which displayed 25% and 100% stroma invasion, respectively [30,122]. Accordingly, these findings demonstrate both *p53* tumor-suppressive function and the varied effects of *p53* at various mutant loci [30,122]. Together, these results highlight the *Apc^Min/+^* mouse model’s significance in the investigation of colon tumorigenesis by establishing the pioneering function of the *Apc* mutation in the emergence of CRC.

The main disadvantages associated with the use of APMM are cost and complexity. These models require specific genetic modifications which can be costly to obtain, and they may not be amenable to certain therapies and treatments due to the inherent variation between individual mouse strains. Additionally, due to the complexity of the disease and the need to replicate multiple tumor characteristics, these models are difficult to design and set up compared to other pre-clinical models [117].

### 4.2. Hereditary Nonpolyposis Colon Cancer Mouse Models (HNPCC)

Germline pathogenic variants in DNA MMR genes cause hereditary non-polyposis CRC, also known as Lynch syndrome (LS), which is one of the most common cancer predisposition syndromes [123]. LS is caused by mutations in the DNA MMR genes *Mlh1*, *Msh2*, *Msh3*, *Msh6*, *Pms1,* and *Pms2* (MutL Homolog 1; MutS Homolog 2, 3, and 6; and Post-Meiotic Segregation Increased 1 and 2; respectively) alone or in combination with a germline mutation in the *Apc* tumor-suppressor gene [116,124].

DNA MMR gene mutations cause chromosomal instability, while *Apc* mutations cause microsatellite instability (MSI). CRC, and LS in particular, can be affected by MSI in the promoter regions of the *Apc*, *TGF-βRIII*, and *Bax* genes [125]. Patients who have a hereditary predisposition to CRC (such as those with FAP or LS) often have severe genetic defects due to germ line mutations in tumor-suppressor genes and DNA MMR genes [126]. Incidence rates for LS are about as high as those for other subtypes of cancer combined (1 to 5%); however, sCRC also exhibits somatic mutations in these genes [126,127].

HNPCC murine models are useful tools for pre-clinical assessment of CRC therapies. These models involve the generation of mice that possess a genetic mutation known as the MMR defect, which is responsible for LS in humans. This genetic mutation, combined with other environmental factors, causes these mice to develop multiple malignant tumors in the colon and rectum, similar to the human condition. HNPCC murine models provide researchers with an in vivo platform to study the underlying biology and pathways associated with LS, as well as evaluate potential therapies.

Examples of HNPCC murine models include *Mlh1*, *Mlh3*, *Msh2*, *Msh6*, and *Pms2* knockouts [128]. The *Mlh1* and *Msh2* knockouts involve the generation of mice lacking either the *Mlh1* or *Msh2* gene, resulting in a defective MMR system which leads to multiple tumors in the colorectum [129]. Similar to *Mlh1*- and *Msh2*-deficient mice, the loss of both *Msh3* and *Msh6* increases gastrointestinal tumors at a much younger age, whereas *Msh3* loss does not increase cancer susceptibility until later in life [130].

Other example of HNPCC murine models include the epithelial cell adhesion molecule (EpCAM) [131,132,133] knockout animals. EpCAM knockout animals lack the epithelial cell adhesion molecule EpCAM, which is delete in patients with LS, resulting in multiple tumors in the colorectal region [131,132,133].

There are also multiple transgenic murine models that have been developed for LS, including the *Kras^G12D+^* and *Pms2* transgenic animal models. The *Kras^G12D+^* expresses a constitutively active mutant form of the *Kras* gene, leading to the formation of numerous malignant tumors in the colorectum and with high lymph node metastases [30,134]. The *Pms2* transgenic mouse model overexpresses the *Pms2* gene and results in the formation of multiple tumors in the colorectum. In Biswas et al. [135] study, an increased intestinal polyp formation of ≈4.5-fold was observed in *Pms2^ki/ki^* mice with heterozygous *APC* mutation (chain-termination mutation in the 15th exon, *Apc^+/−^)* compared to *Apc^+/−^* or *Pms2^ki/+^*;*Apc^+/−^* mice. Accumulated MSI is an indicative sign of MMR deficiency, and this was also demonstrated in *Pms2^ki/ki^* intestinal adenomas.

The use of HNPCC murine models in CRC research has numerous advantages. These models provide an in vivo platform to study the biological pathways associated with LS and the formation of tumors. Additionally, they can be used to evaluate potential therapies in pre-clinical studies, as well as gain insight into the development of resistant tumor subtypes.

They can also be used to better understand drug delivery mechanisms and to assess response to therapeutics. Other advantages include the ability to assess gene expression profiles, identify new biomarkers, and gain insight into the molecular mechanisms of cancer progression. Moreover, these models can provide a platform for testing novel combinations of targeted therapies and immunotherapies, as well as studying the mechanisms of drug resistance.

Finally, they can also be used to assess recommendations of dietary modification, environmental factors, and lifestyle habits that may influence cancer progression; however, these models are expensive and time-consuming to create, and there can be variability in the phenotypes of the mice due to environmental factors. Additionally, the results obtained from these models might not be directly applicable to humans due to differences in biological pathways and genetic backgrounds. Thus, there is still a limited understanding of the role of genetic mutations in the development of LS, making it difficult to accurately predict the outcome of treatments using these models.

**Table 2:** Overview of Genetically Engineered Murine Models (GEMMs) in CRC research.

GEMM	Outcome(s)	Advantages	Disadvantages	Ref.
All	Evaluate the role of genes involved in carcinogenesis; Studies of chemoprevention and therapeutic agents; Assessing the influence of carcinogens; Lifestyle/dietary influence on tumor formation.	Genetic event is known; In situ tumor development; Reproduces early stages of oncogenesis; Modified gene is expressed on physiologic level; Tumor cells and stroma are from the same specie; Intact immune system.	Limited options for non-invasive imaging (would need CT/MRI capability); Expensive and time consuming to develop; Only partial replication of the human tumoral morphology and physiology; Secondary mutations are different from the human tumors; Low metastases rate.	[50,136]
*Apc^580S^*	Adenoma formation in the distal rectum in most of the *Apc ^580S^* homozygotes within 4 weeks after infection by rectal infusion with recombinant adenoviruses encoding the Cre recombinase. In total, 50% of animals show invasive adenocarcinoma after 1 year without lymphatic or distant metastases.	Useful for studying the mechanism of CRC development and to test therapeutic or chemopreventative agents.	Only effective in *Apc ^580S/580S^* mice and not *Apc ^580S/+^*, an outcome that reflects the poor ability of the approach to influence the proliferating cells at the crypt base.	[137]
*CAC; APC^580S/+^*	Adenomatous lesions in the distal colon; DSS treatment increased the incidence and number of tumors, and this occurred predominantly in distal colon.	Mimics the tissue and cellular environment of heritable cancers such as FAP and LS.	Early CRC development may limit the ability to test therapeutic or chemopreventative agents; increased animal numbers for CRC studies.	[138]
*Apc^Min/+^ Mom1^R/R^ P53^−/−^*	*p53* deficiency increases intestinal adenoma multiplicity and malignancy.	*p53*-deficient tumors studies	Short lifespan (122 days).	[139]
*Apc^Min/+^ Mom1^R/s^ P53^−/−^*				
*K-Ras^G12D^*	Adenocarcinomas expressing invariably exhibit uniform high-grade dysplasia	*KRAS* signaling pathway studies	Do not develop metastases.	[140]
*Pik3ca^H1047R^*	Develop invasive adenocarcinomas strikingly similar to invasive adenocarcinomas found in human CRC.	PI3K/AKT/mTOR pathway therapeutic studies	Late CRC	[141]
*Msh2^−/−^*	Development of colorectal tumors with defects in DNA mismatch repair.	Model of LS (3% of all CRCs)	*Msh2* mutation in all cells of body and mice are predisposed to lymphomas.	[142]
*Smad4^TKO^*	Development of colorectal tumors with loss of function mutations in the tumor-suppressor gene *Smad4.*	IFN-γ expression correlates with the onset of spontaneous CAC by 6 months of age.	Do not develop metastases.	[143]
*Apc^CKO^/LSL-Kras*	Cre-mediated knockout of *Apc* and *Kras^G12D^* activation by surgical application of AdenoCre to the colonic epithelium leads to tumor formation after 3 weeks and adenocarcinomas with 20% liver metastases after 20 weeks.	FAP and LS genetic mutations are present in the germline; mTOR Pathway and metastatic model.	20–24 weeks for metastases development.	[144]
*Villin-Cre/K-ras^G12Dint^/Ink4a/Arf^−/−^*	Most invasive adenocarcinomas (79%) progress within 12 weeks, and 60% of these tumors metastasize to the lungs.	Use tissue specific promoters in intestinal mucosa to target gene knockout; Some invasive adenocarcinomas seen can be used to target specific tumor-suppressor or oncogenes.	Requires rectal instillation of recombinant adenovirus expressing Cre.	[145]
*Villin-Cre; LSL-Kras^G12D/+^*				
*Villin-Cre; Kras^G12Dint^*				
Lgr5^CreERT2^	Hyperproliferating intestinal adenomas were formed 4 weeks after tamoxifen injection.	CDX models (HCT-116 or SW480 cells); Wnt/*β*- catenin pathway	Do not develop metastases.	[146]
*β*-catenin^exon3^				
Rosa26^LSL-rtta-ires-EGFP^				
TRE-Spdef				
*Apc^1638N/+^+* AOM	A 6-fold increase in colonic tumor formation compared to *Apc ^Min/+^* mice; higher incidence of colonic adenocarcinomas.	Increased the tumor burden in the colon; Suitable and straightforward model to study the influence of immune cells and chemokines on colon carcinogenesis.	Do not develop metastases.	[120]
*Apc ^Min/+^ +* PhIP	Increased tumor development by 2- to 3-fold compared to *Apc ^Min/+^* mice	Ideal gene expression for FAP studies	Do not develop metastases; Most of the tumors are in the small intestine.	[147]
*Apc^Δ716^ Tgfbr2^flox/flox^*; *villin-CreER +* DSS (2%*)*	TGF-signaling disruption include the development of adenocarcinomas with a local invasion pattern	Ideal for CAC CRC studies	No metastases reported.	[148]
*Apc^Δ716^ Kras^G12D^*	Increased multiplicity of intestinal tumors	Metastatic model; PDOX model; Efficient metastases by Wnt activation, *Kras* activation, and TGFβ suppression combination.	No spontaneous metastases.	[149]
*Apc^Δ716^ Trp53^R270H^*	Developed adenocarcinomas with invasion to submucosa or deeper			
*Apc^Δ716^Kras^G12D^Fbxw7^−/−^*	Distinct histologic type and accelerated tumorigenesis			
*Apc^Δ716^Kras^G12D^Tgfbr^−/−^*	Efficient liver metastases			
*Dpc4^+/−^: Apc^+/Δ716^*	Submucosal infiltration and a progression from adenoma to carcinoma can be seen in the small intestine and colon of *Dpc4* and *Apc^Δ716^*cis-compound heterozygote mice	Ideal for FAP CRC studies (Histological features of tumors are identical)	Do not develop metastases.	[150]
*Fen1^null^/Apc^1638N^*	Increased intestinal tumor malignancy via MSI comparatively to *Apc^1638N^* mice	FAP and LS studies with *Fen1* gene	Do not develop metastases.	[151]
*Fbw7^flox/flox^*; *P53^flox/flox^*; *Villin-Cre*	Allografts derived from tumors with a double deletion of *Fbw7* and *p53* develop into highly malignant adenocarcinomas with a high rate of metastases	Important tool for future studies of the pathogenesis and treatment of metastatic and chromosomally unstable CRC.	Long latency period (up to 101 weeks)	[152]
*AhCre^+/T^*; *Kras^+/LSLV12^*, *Apc^+/fl^*	Although *Kras^V12^* mutation does not affect the intestinal epithelium, it accelerates tumorigenesis when combined with *Apc* loss. Invasive adenocarcinomas make up 17% of all tumors	Suitable for Raf-MEK-ERK pathway studies	Do not develop metastases.	[153]
*Pms2 ^ki/ki^*	A ∼4.5-fold increase in intestinal polyp formation compared to *Apc^+/^^−^* or *Pms2^ki/+^*; *Apc^+/^^−^* mice	LS studies with MMR genes; Suppression of de novo splice site.	Do not develop metastases.	[135]
*BRAF-V600E*	Promotes rapid serrated tumor development and progression and assesses the role of *Smad4* in early-stage serrated tumorigenesis	Oncogenic β-catenin mutations (combinations of *Ctnnb1*, *Braf*, and *Smad4*) drive rapid serrated dysplasia formation.	Do not develop metastases.	[154]

CAC—colitis-associated colorectal cancer; CRC—colorectal cancer; CDX—cell-derived xenografts; FAP—familial adenomatous polyposis; LS—Lynch syndrome; MMR—mismatch repair.

## 5. Transplant and Metastatic Murine Models

In contrast to xenogeneic grafts, syngeneic tumor transplantation is characterized by the engraftment of tumor tissue or cancer cell lines inside the same murine strain. Herein, it is also possible to discriminate between orthotopic and heterotopic models.

The three cornerstones of Transplant Metastatic Models (TMMs) are host, xenograft, and methods of transplantation. The host has undergone numerous alterations from the first generation to the fourth generation over the course of many years of continual development and growth. Specifically, immunocompetent, genetically immunodeficient, new combination immunodeficient, and humanized murine models make up the first, second, third, and fourth generations of hosts, respectively.

Metastasis the leading cause of death in patients with CRC [155]; therefore, it is essential to employ murine models to replicate the clinical characteristics to study the underlying mechanism and find an effective treatment for. Although significant CRC murine models have been developed, animals that can develop metastatic characteristics remain scarce.

In the following section, several transplant and metastases murine models will be discussed.

### 5.1. Transplant Murine Models (TMMs)

TMMs has been extensively used in several areas of CRC therapy as a bridge to clinical use. Each TMM has distinct qualities, yet each model also has limitations. For the creation of TMMs, cell-derived xenografts (CDX), patient-derived xenografts (PDX), and/or patient-derived organoids xenografts (PDOX) are generally employed. Tumor materials are mostly transplanted into the host by s.c., intrasplenic, or orthotopic pathways to generate the TMMs. For instance, CDX models do not accurately represent patients’ treatment responses, which has a relatively poor clinical approval rate for cancer drugs (less than 15%) [156]. Through extensive pharmacological testing, s.c. transplantation models have little prognostic value for human clinical response [157]. The orthotopic PDX models, in contrast, continue to exhibit the highest concordance of treatment responses across human patients and murine models, hence confirming their use as the best screening platform for the assessment of CRC anticancer drugs [158].

**Figure 2:**
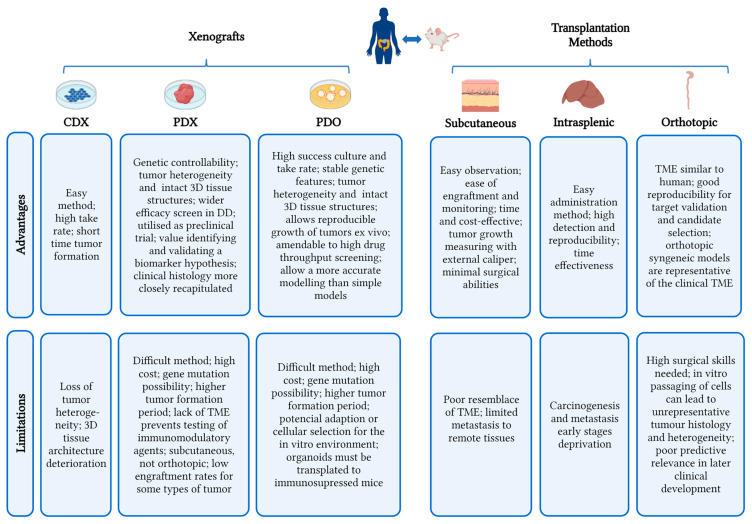
Summary of the characteristics of transplant murine models’ xenografts and transplantation methods. Created with BioRender.com, accessed on 29 January 2023. DD—drug discovery; TME—tumor microenvironment.

At the present, the creation of biomarkers, pharmacological testing, and surgical modeling are the main applications of TMMs. Figure 2 summarizes the main characteristics of TMMs.

#### 5.1.1. Cell-Derived Xenografts (CDX) Models

From cell line banks all across the world, more than 100 cell lines have been established as CRC cell lines. To find underlying mechanisms and develop cancer drugs, CDX models were created by implanting cancer cell lines into immunodeficient animals [158]. When human CRC cells are s.c. injected into an immunocompromised mouse, the injection site frequently develops a tumor. The conventionally naked (athymic) and severe combined immunodeficient (SCID) mouse strains, which lack T lymphocytes or both B and T lymphocytes, respectively, are frequently utilized. In contrast to SCID mice, NOD/SCID mice also lack NK cells [159].

This model is commonly used in CRC research and DD due to its low level of technical expertise, ease with which tumor growth can be observed, affordable cost of maintaining colonies, high yield production and tolerable tumor latency [160]; however, due to the loss of original inheritance and the absence of pertinent tumor microenvironment (TME) components during in vitro passage, this model is unable to reproduce the tumor genetic heterogeneity of the initial tumor [161]. Additionally, repeated passaging with enrichment for particular subclones may result in changes at the genetic and epigenetic levels [162].

The C57BL/6-derived adenocarcinoma line MC38 and the Balb/c-derived lymphoma line CT26 are commonly used in studies of syngeneic s.c. transplantation [160]. Due to their high mutation rates, these cells have been proven to be effective preclinical models of human tumors; however, the success of orthotopic engraftment of MC38 tumors has varied greatly between studies, in contrast to the high fidelity with which CT26 cells have been implanted [163].

Concerning human cell lines, HCT-116 is one of the most popular CRC cell lines [117,164,165,166,167,168,169,170,171,172,173,174,175,176]. Beside HCT-116 cell line, several other cell lines are utilized in CRC research, such as HT-29 [3,177,178,179,180,181,182], SW620 [183,184,185,186], T84 [179,187], LoVo [188,189], LS174T [190,191,192,193], DLD-1 [194,195,196,197], and SW480 [85,146,188].

Subcutaneous CRC cell line xenograft has several advantages and drawbacks. The main benefits of injecting cells into immune deficient mice are their low cost, rapid tumor growth, well-characterized cell lines, ease of genetic manipulation and model accessibility [116]. However, besides representing the disease at an advanced stage it has undergone significant clonal selection and rarely metastasizes [116].

In orthotopic xenografts of CRC cell lines, the injection of cells into intestinal serosa of immune deficient mice also has their benefits and disadvantages. The benefits of this model are similar to the s.c. model with the difference being a more natural microenvironment for CRC cells and that some cell lines metastasize to the liver (e.g., HCT-116 or HT-29). The disadvantages are similar as in s.c. model with the exception of a surgical requirement to implant cells [116]. Orthotopic implantation of human CRC cells such as HT-29, SW620, HCT-116, and SW480 is commonly performed using immunodeficient humanized mice, such as severe combined immunodeficient, Rag1, or nude mice; however, the inability to study adaptive immunity, cytotoxic T cells, or checkpoint blockade therapies is a major drawback of these models for immuno-oncology studies [163].

Syngraft/Isograft models involve transferring 1–2 mm mouse tumor fragments or mouse tumor cell lines to a genetically identical inbred, immune-competent mouse. These models have strengths such as no species mismatch between tumor and stromal cells, and an intact immune system that enables immunotherapeutic studies; however, they are labor intensive and time consuming and do not use human cell lines, making them the main weaknesses [116].

In order to expand our understanding of tumor biology and better identify innovative therapeutics for cancer treatment, PDX models have been developed in order to get around the limitations of the CDX model [172,198,199,200].

#### 5.1.2. Patient-Derived Xenograft (PDX) Models

The PDX model is a murine tumor model created by grafting human tumors onto immunodeficient mice. It has been demonstrated to be a useful tool for studying the biology of tumors and assessing the effectiveness of anticancer agents in a variety of tumor types [201,202]. Drug evaluation outcomes are most comparable to clinical situations, and the type of immunodeficient mice used and the delivery method affect the rate of engraftment [201,202]. Additionally, it has been demonstrated that PDX models preserve the heterogeneity of the underlying tumor in CRC [203,204,205,206]. To facilitate engraftment, monitoring and resecting the tumor s.c. implantation is the most common [13,156,207]; however, this model was created using tumor tissue cells that were extracted through enzymatic digestion in multiple investigations [208].

In other studies, researchers created CRC PDX8models orthotopically with endogenous metastases that can travel to the lungs and liver similarly to a patient’s main tumor. To the best of our knowledge, metastases to other organs do not occur in s.c. engraftment [24,199,204,208]. The highest rate of engraftment (60–100%) in CRC is seen in PDX models using Balb/c nude mice (100%) as hosts. Nevertheless, NSG and NOD/SCID mice are also frequently used [206,209]. Surgical specimens are the most frequently employed original source because the amount of initial tumor material has a significant impact on the success of PDX engraftment [210].

There are important gene mutations including *Kras*, *Braf* (v-Raf murine sarcoma viral oncogene homolog B), and *PIK3CA* of the primary tumor that are still present in PDX CRC models, in addition to gene expression, copy number alterations, and MSI [156].

PDX is rich in stromal component compared to 2D cultivated cancer cell lines, which may be advantageous for research on the interactions between cancer cells and TME. It has been demonstrated that PDX maintains the global gene–expression patterns, mutational status, metastatic potentials, histological differentiation, and histopathological subtypes of the human donor tumor [156,210,211].

Numerous studies have used the CRC PDX model to assess the effectiveness of immunotherapy drugs and other systemic chemotherapeutic agents [212,213,214], as well as to identify drugs and biomarkers [166,198], produce cell lines [215,216], create colospheric structures [217], and learn more about tumor biology [218]. As a result, it can be used to create individualized cancer treatments; however, there are certain restrictions on using PDX, such as a lengthy engraftment phase, usually lasting 4–8 months. Therefore, employing the PDX model to offer patients with fast drug screening findings can be difficult [219]. Further, the poor cryopreservation and reanimation efficiency of PDX raises the possibility of losing valuable tumor samples [220].

Recent research has shown that the humanized mouse model is useful for studying the human immune system and evaluating the response to immunotherapeutic drugs. But there are ethical concerns with conducting experiments on humanized mice, such as the need for a tissue bank consisting of various types of human leukocytes and human hematopoietic cells obtained from bone marrow, umbilical cord blood and fetal organs [221,222,223]. Chimera studies are often considered unethical due to the crossing of species barriers [224].

PDX orthotopic models provide a strong framework for investigating the biology of metastases and therapeutic response in CRC. Nevertheless, this model has a limited capacity and reproducibility due to the technical ability required for orthotopic implantation. In recent years, it has also been possible to create PDXs that sustain tumorigenicity in mice by using fluid from malignant ascites or pleural effusions, circulating tumor cells (CTCs), or both [204,225,226,227]. Ex vivo cultivated CTCs have been demonstrated to retain their tumorigenic capacity in CRC [228]. As a result, CTC-derived PDX models show potential for the analysis of tumor genomic evolution and the assessment of tumor responses to new therapeutics.

#### 5.1.3. Patient-Derived Organoid Xenograft (PDOX) Models

It is known that PDX model is a time-consuming and relatively expensive task but the patient-derived organoids (PDOs) offer a potential solution for that issues. PDOs are developed from isolated organ progenitor cells or patient stem cells collections that originate clusters of 3D cultivated multicellular aggregates [13,229]. Evidence suggests that PDOs maintain both the tissue functions and the properties of the parent matrix. Furthermore, PDOs faithfully mimic in vivo tissues during homeostasis and diseases such as CRC. This model is simple to maintain, and it can genetically editable. It has been demonstrated that PDOs retain the genetic, transcriptomic and histological traits of the parental tumors [229].

On the other hand, traditional PDOs frequently only contain cancer cells and lack TME constituents, including fibroblasts, endothelial cells, and immune cells, among others. As a result, efforts are still being made to recreate the parental tumors’ microenvironment by including TME components into organoids [13,230,231]. PDOs have some drawbacks, including having a protracted culture cycle, a single-cell source, variable culture conditions, and being time-consuming and a technically difficult model [219]; however, being the most recent source of xenografts with exceptional fidelity and adaptability in the CRC model, PDOs are still seen as a major advance in the study of cancer biology and therapeutic response, after the PDX model [231].

PDOX murine models have emerged as a useful tool for pre-clinical evaluation of CRC therapies, surpassing most of the limitations of CDX, PDX and PDO models. This model involves the generation of a PDOX cell line from a patients’ tumor, which is then transplanted into a humanized mouse [222] or a mouse with an immunodeficient background. This model has been shown to accurately replicate both the histology and biology of the patients’ tumor and to retain driver mutations present in the original tumor. This mouse background is preferred, as it eliminates any potential rejection of the tumor cells due to its lack of an adaptive immune system while still retaining the necessary cell signaling pathways to support the growth of the tumor cells [232].

A key advantage of the PDOX model is its ability to accurately replicate both the histology and biology of the patients’ tumor, as well as retain driver mutations present in the original tumor. This allows for realistic assessment of drug efficacy and toxicities in an in vivo model that closely resembles the clinical situation. Another advantage is PDOX high success rate of production from primary CRC tissue (up to 90%). PDOX implantation provides a solid foundation for more accurate CRC murine models due to the tumor formation rate of 60% and 100% for implantation into the colon wall and cecal, respectively [13]. In addition, the technique allows researchers to study drug resistance and cancer metastases, which are two major factors in determining the overall efficacy of treatments. Moreover, PDOX models have been employed to evaluate the potential combination therapy strategies to treat CRC, by testing the efficacy of individual therapies in combination with each other. Finally, this PDOX model is suitable for pre-clinical evaluation of CRC therapies, providing researchers with a platform to effectively analyze and test [233].

The main drawbacks of PDOX murine models in CRC are the complexity and cost associated with setting up the model. As it requires reprogramming the patient’s tumor and transplanting it into a mouse background, the procedure can be quite costly. Nevertheless, when comparing against PDX models, PDOX are less costly to establish and have a high throughput [231]. Additionally, as this procedure is relatively new, there is still a lack of validation and standardization protocols regarding PDOX technology. Finally, as these models are based on individual patients, they may not be suitable for providing generalizable results across different patient populations.

### 5.2. Metastases Models

The key factor contributing to CRC patients’ high mortality rate is distant metastases [234,235]. Therefore, revealing biomarkers that predict drug response and identifying patients that are most likely to benefit from a specific treatment is crucial.

Animal models should be carefully chosen to closely resemble the molecular, histopathological and etiological features of the donor tumors [236]. Studies on metastases have made extensive use of PDX, GEMMs, and PDOX models (Table 3) [237]. Xenograft models are ideal for testing new therapies, but they remove the protective role of the immune system in disease development. It is possible to avoid some or all the steps necessary for metastases formation by injecting tumor cells directly into the cecal or colonic wall, or even into the bloodstream. GEMMs are widely used in studies of carcinogenic progression and the mechanisms of individual cancer-related genes, but they can be costly and have a long latency.

Research groups have developed orthotopic CRC PDX models, which preserve the TME necessary to investigate tumor cells with metastatic potential [204,216,238,239]. In Rashidi et al. [240] study, all mice implanted with a tumor had liver metastases within 10 days, and lymph nodes draining to the liver showed metastases 19 days after implantation; however, the main drawbacks of metastatic PDX models are that it is time-consuming, technically difficult and high-cost [219].

**Table 3:** The most representative studies of CRC transplant and metastases models.

Model	Predominant Histopathology	Metastases and Main Location	Ref.
**Carcinogen-induced Models(CIM)**			
Tp53^ΔIEC^ + AOM	Adenocarcinoma	Lymph nodes	[241]
LSL-Kras^G12D^/+; p53 ^flox/flox+^sgApc-Cas9-Cre	Adenocarcinoma	Lymph nodes and liver	[242]
**Genetically Engineered Models (GEMMs)**			
Apc^CKO/CKO^LSL-G12D; Kras ^tm4tyj/+^	Adenocarcinoma	Lymph nodes and liver	[144]
Apc^Lox/Lox^; p53^Lox/Lox^; Tet-O-LSL-Kras^G12D^; VillinCre^ERT2^	Adenocarcinoma	Lymph nodes, liver, and lungs	[243]
Villin-Cre^ERT2^ *Apc* ^fl/fl^	Adenocarcinoma	Lymph nodes	[179]
LSL-KRAS^G12V^/APC^flox/flox^	Adenocarcinoma	Lymph nodes and liver	[244]
**Cell-derived Xenografts** (**CDXs)**			
NSG mice + HT29^p53-mut^/LUC cells	Adenocarcinoma	Lymph nodes, liver, lungs, and bone marrow.	[3]
Balb/c (i.c.) + CT-26 cells	Carcinoma	No metastases	[245]
NOD/SCID (i.c.) + HCT-116 cells	Adenocarcinoma	Liver	[234]
Balb/c nude mice (s.c.) + HCT15 cells	Adenocarcinoma	NR	[246]
Balb/c nude mice (s.c.) + HCT-116 cells	Adenocarcinoma	NR	[247]
Balb/c nude mice (i.v.) +HCT-116-Luc cells	Adenocarcinoma	Lungs	[248]
C57BL/6J mice (s.c.) + MC38 cells		NR	
*Traj18*^−/−^ (s.c., i.c.) + MC38 cells	Adenocarcinoma	NR	[249]
*CD1d^−/−^* (s.c., i.c.) + MC38 cells	Adenocarcinoma	NR	
NSG mice (i.c.; i.s.) + SW480 cells	Adenocarcinoma	Liver	[250]
NSG mice (i.c.; i.s.) + SW620 cells	Adenocarcinoma	Liver	[250]
Balb/c nude mice (i.c.) + SW620 cells	NR	Liver	[187]
**Patient-derived Xenografts (PDX)**			
NSG mice (i.s.)	Adenocarcinoma and carcinoma	Lymph nodes, liver and lungs.	[251]
Balb/c mice (i.s.)	Adenocarcinoma	Liver	[240]
Balb/c nude mice (i.c.)	NR	Liver	[187]
Balb/c nude mice (s.c.)	Adenocarcinoma	NR	[248]
NCG mice (i.v.)	Adenomas and carcinomas	Liver and lungs.	[252]
NOD/SCID mice (i.c.)	Adenocarcinoma	Lungs	[253]
NOD/SCID mice (s.c.)	Carcinoma	NR	[254]
NCG mice (s.c.)	Adenocarcinoma	NR	[255]
**Patient-derived Organoids Xenografts** (**PDOXs)**			
Balb/c-nu mice (i.s.)	Macrometastatic colonies	Liver and lungs.	[236]
NOG mice (i.s.)	Micro- and macrometastatic colonies	Liver	[256]
NSG mice (i.s.)	Macrometastatic colonies	Liver	[257]
NSG mice (s.c.; i.c.; i.s.)	Micro- and macrometastatic colonies	Liver	[250]

i.s.—intrasplenic; s.c.—subcutaneous; i.c.—intra-cecal; i.v.—intravenous; NR—not reported.

GEMMs of CRC can spread to the liver, as demonstrated by a study [244] in which LSL- *KRAS^G12V^/APC^flox/flox^* mice and an AdenoCre were injected into the colon. This causes the activation of oncogenic *KRAS^G12V^*, loss of *Apc* tumor suppressor, development of sCRC, and liver metastases [244,258]. 

GEMMs are useful for studying the role of individual genetic mutations in the carcinogenic process, promoting tumor progression and liver metastases while limiting over-growth of cancer cells. They better depict the dynamics between tumor cells and their microenvironments throughout tumor progression than TMMs [259]. Furthermore, they are useful for assessing the earliest stages of tumor development [24,259]; however, mutations in genes can cause embryonic lethality, developmental defects, or sterility prior to the development of liver metastases in GEMMs. Thus, due to the slow progression and low incidence of metastases in GEMMs, it can be challenging to assess therapeutic responses [24,28,136].

In vivo manipulation of 3D CRC organoids has recently been described, with cecal or colonic implantation of these structures [236,237]. CRC and liver metastases models have been established by transplanting PDOXs with multiple cancer-related mutations into the colon, renal capsule, and spleen of mice [236,237]; however, neither tumor invasion into the muscularis propria nor tumor extravasation into the circulation through the colon serosa can be studied in these ectopic transplantation models [43,260]. To examine primary cancers and liver metastases, some research groups used orthotopic transplantation to place PDOXs into the colonic or rectal mucosa of NRGA-immunodeficient mice (PDOXwE), which was then subcultured in Balb/c mice [261,262]. Using gene-editing techniques, PDOXs can be engineered for desired mutations, a process that is much quicker than creating germline GEMMs.

PDOXs can also have fluorescent labels and other features added to them with relative ease [13]. Through xenotransplantation into the kidney subcapsules of immunodeficient (NOG) mice, Fujii et al. generated matched pairs of primary and metastatic organoids from CRC patients [256]. Organoids derived from CRC metastases in this model were more able to metastasize than their primary tumor counterparts. Orthotopic implantation of tumor organoids is preferable to the germline GEMM for pre-clinical study because it ensures that all mice in the same experiment have tumors of the same number and similar volume. These benefits ensure that the orthotopic CRC model based on organoids will be the most sought-after and useful model for future preclinical research [28].

CRISPR-Cas9 technology, for example, has added flexibility to genomic editing and has been heralded as a highly effective tool for achieving metastatic disease, particularly when combined with CRC organoids [136,262].

## 6. Meeting the Criteria for a Successful Murine Model for Colorectal Cancer Investigation

Previous murine models for CRC were compared in terms of key criteria to assess their potential for a successful CRC investigation and their potential translation into preclinical and clinical settings. This general comparison is depicted in Table 4, and it was based on authors’ opinion.

## 7. Future Perspectives

The response of the tumor to anticancer drugs is highly variable, making it essential to comprehend the role of a heterogeneous TME in order to effectively manage treatment. For effective therapy management, understanding the role of a heterogeneous TME is essential.

It is known that highly translational cancer models are becoming increasingly important in the field of precision medicine. 

The use of animal models always following the 3R principles (replacement, reduction, and refinement) is crucial but other models can and should be also used to complement that information. This indicates that in vitro and in silico models must also be improved in parallel to in vivo models. Organs-on-chips (OoCs) are one example of a key tool for this purpose. OoC platforms recreate key features of the TME in vitro [175]. An OoC model combines 2D and 3D cell-culture advancements with artificial organs that mimics the most typical sites of metastatic spread [263]. In addition, Guinney et al. highlight the importance of computational models in cancer science and the potential of bioinformatics research [264]. The relationship between treatment response and molecular subtypes has been partially shown by retrospective analysis of clinical trial samples [265].

A strategy to integrate and analyze the vast amount of data is required due to the constantly expanding knowledge of cancer pathways and their interaction on the one hand, and the rising interindividual complexity of tumors on the other [266,267]. Data mining, pattern recognition, machine learning, and network approaches are some examples of in silico models/techniques that can predict the behavior of virtual CRC cells [268], identify new biomarkers [269], identify unknown driver mutations [270], reveal genetic patterns linked to survival [271], and identify potential compounds [272,273]; however, they still have a long way to go before they can accurately forecast how a new compound will affect a patients’ treatment response [273].

## 8. Conclusions

Murine models of CRC have yielded insights into pathogenesis mechanisms, tools for drug discovery, validation of novel therapeutic targets and a predictive platform for testing new preventative and therapeutic strategies. CIMs, GEMMs, TMMs and metastatic models have been used to study various aspects of CRC. 

In this search, we observed that there is still not only one model to fully address this disease. In case of CIMs, these models are a powerful tool for understanding the development and progression of the CRC. Additionally, Cre-LoxP technology and TMMs have been used to advance our understanding of CRC but are limited in their ability to accurately reflect the complexity of the human immune system. PDOX models are challenging to use to investigate the impact of immune TME components on cancer progression, but more research is needed to confirm these facts.

To sum up, there is still a need for a model that can correctly reflect the pathophysiology of CRC. In the meanwhile, murine CRC models will continue to be an important tool in advancing our understanding and treatment of this disease.

## Figures and Tables

**Table 4:** Murine models performance in CRC research.

	Models	CIMs					GEMMs		TMMs			
Features		DMH	AOM	HCA	DMAB	AS	APMM	HNPCC	CDX	PDX	PDOX	MM
Complexity		+	+	+	+	+	+++	+++	++ ^a^	++	+++	+++
Time consuming		++	++	++	++	++	+++	+++	++ ^a^	+++	+++	++
Cost		+	+	+	+	+	+++	+++	++	+++	+++	+++
Surgical skills		+	+	+	+	+	++	++	++ ^a^	+++	+++	+++
Translational models		++	++	+	+	+	+++	+++	++	++	+++	++
Tumor heterogeneity		+++	+++	++	++	++	++	++	++	+++	+++	++
Tumor microenvironment		+++	+++	+	+	+	+++	+++	+	+ ^c^	++	++
Engraftment rate		+++	+++	++	++	++	++	++	++	++	+++	++
Metastases		+	+	+	+	+	++	++	++ ^a^	++	++	+++
Precision medicine		++	++	+	+	+	++	++	+	++	+++	++
Chemotherapy studies		+++	+++	+	+	+	++	++	+++	++	+++	+++
Immuno-oncology		++	++	+	+	+	+++	+++	++ ^d^	+ ^b^	++	+
High-throughput omics		+	+	+	+	+	++	++	+	++	+++	+
Drug discovery		+++	+++	+	+	++	++	++	+++	++	++	++
Biomarker discovery		++	++	+	+	+	+++	+++	++	+++	+++	++

Classification: + low; ++ moderate; +++ high; ++ ^a^ in orthotopic and isograft models (+ in s.c. models); + ^b^ in humanized PDX models; + ^c^ immunocompromised mice (++ in GEMMs and humanized mice); ++ ^d^ in s.c. and orthotopic model (++ in isograft model) when using human cell lines; CIMs—cancer-inducing murine models; TMMs—transplant murine models; GEMMs—genetically engineered murine models; DMH—1,2-dimethylhydrazine; AOM—azoxymethane; DMAB—3,2-dimethyl-4-aminobiphenyl; HCA—heterocyclic amines; AS—alkylating substances; APMM—adenomatous polyposis mouse models; HNPCC—Hereditary Nonpolyposis Colon Cancer Mouse Models; CDX—Cell-Derived Xenografts Models; PDX—Patient-Derived Xenograft Models; MM—metastases models.

## Supplementary Materials

The following supporting information can be downloaded at: https://www.mdpi.com/article/10.3390/cancers15092570/s1, Table S1: Colorectal cancer etiology.

## Abbreviations

ACF	Aberrant crypt foci
AOM	Azoxymethane
APMM	Adenomatous Polyposis Mouse Models
b.w.	Body weight
CAC	Colitis-associated CRC
CD	Crohn’s disease
CDX	Cell-derived xenografts
CIMs	Carcinogen-induced models
CRC	Colorectal cancer
CTCs	Circulating tumor cells
DMAB	3,2-dimethyl-4-aminobiphenyl
DMH	1,2-dimethylhydrazine
DSS	dextran sulphate sodium
FAP	Familial adenomatous polyposis
GEMMs	Genetically Engineered Murine Models
H	Hours
HCAs	Heterocyclic amines
HNPCC	Hereditary Nonpolyposis Colon Cancer Mouse Models
IBD	Inflammatory bowel disease
i.c.	Intra-caecal
i.g.	Intragastric gavage
i.m.	Intramuscular
i.p.	Intraperitoneal
i.r.	Intrarectal
i.v.	Intravenous
IQ	2-amino-3-methylimidazo[4,5-*f*]quinoline
LS	Lynch syndrome
MAM	Methylazoxymethanol
MM	Metastatic Models
MNNG	N-methyl-N-nitro-N-nitrosoguanidine
MNU	Methylnitrosourea
mos	Months
MSI	Microsatellite instability
NA	Nuclear aberration
NF-κB	nuclear factor kappa B
NR	Not reported
OoC	Organs-on-chips
o.g.	Oral gavage
PDO	Patient-derived organoids
PDOX	Patient-derived organoid xenografts
PDX	Patient-derived xenografts
PhIP	2-amino-1-methyl-6-phenylimidazo [4,5-b] pyridine
s.c.	Subcutaneous
sCRC	Sporadic colorectal cancer
TME	Tumor microenvironment
TMMs	Transplant metastatic models
TNBS	2,4,6-Trinitrobenzenesulfonic acid
UC	Ulcerative colitis
wk	Week
wks	Weeks

## References

[1] GLOBOCAN (2020). Colorectal Cancer Incidence in The World. Glob. Cancer Obs..

[2] Rabeneck L., Chiu H.M., Senore C. (2020). International Perspective on the Burden of Colorectal Cancer and Public Health Effects. Gastroenterology.

[3] Agarwal S., Behring M., Kim H.-G., Bajpai P., Chakravarthi B.V.S.K., Gupta N., Elkholy A., Al Diffalha S., Varambally S., Manne U. (2020). Targeting P4HA1 with a Small Molecule Inhibitor in a Colorectal Cancer PDX Model. Transl. Oncol..

[4] American Cancer Society Colorectal Cancer Early Detection, Diagnosis, and Staging. Cancer.org. https://www.cancer.org/content/dam/CRC/PDF/Public/8606.00.pdf.

[5] Percario R., Panaccio P., Francesco F., Grottola T., Sebastiano P.D.I. (2021). The Complex Network between Inflammation and Colorectal Cancer: A Systematic Review of the Literature. Cancers.

[6] Ahmad Kendong S.M., Raja Ali R.A., Nawawi K.N.M., Ahmad H.F., Mokhtar N.M. (2021). Gut Dysbiosis and Intestinal Barrier Dysfunction: Potential Explanation for Early-Onset Colorectal Cancer. Front. Cell. Infect. Microbiol..

[7] Alzahrani Mohammad S., Al Doghaither Abdulaziz H., Al-Ghafari Badr A. (2021). General insight into cancer: An overview of colorectal cancer (Review). Mol. Clin. Oncol..

[8] Nguyen L.H., Goel A., Chung D.C. (2020). Pathways of Colorectal Carcinogenesis. Gastroenterology.

[9] Taleban S., Elquza E., Gower-Rousseau C., Peyrin-Biroulet L. (2016). Cancer and inflammatory bowel disease in the elderly. Dig. Liver Dis..

[10] Direito R., Lima A., Rocha J., Ferreira R.B., Mota J., Rebelo P., Fernandes A., Pinto R., Alves P., Bronze R. (2017). Dyospiros kaki phenolics inhibit colitis and colon cancer cell proliferation, but not gelatinase activities. J. Nutr. Biochem..

[11] Frigerio S., Lartey D.A., D’Haens G.R., Grootjans J. (2021). The Role of the Immune System in IBD-Associated Colorectal Cancer: From Pro to Anti-Tumorigenic Mechanisms. Int. J. Mol. Sci..

[12] Marabotto E., Kayali S., Buccilli S., Levo F., Bodini G., Giannini E.G., Savarino V., Savarino E.V. (2022). Colorectal Cancer in Inflammatory Bowel Diseases: Epidemiology and Prevention: A Review. Cancers.

[13] Yang Y.S., Liu C.Y., Wen D., Gao D.Z., Lin S., He H.F., Zhao X.F. (2022). Recent advances in the development of transplanted colorectal cancer mouse models. Transl. Res..

[14] Lotfollahzadeh S., Recio-Boiles A., Cagir B. (2022). Colon Cancer.

[15] Lannagan T.R., Jackstadt R., Leedham S.J., Sansom O.J. (2021). Advances in colon cancer research: In vitro and animal models. Curr. Opin. Genet. Dev..

[16] Tsitskari M., Filippiadis D., Kostantos C., Palialexis K., Zavridis P., Kelekis N., Brountzos E. (2019). The role of interventional oncology in the treatment of colorectal cancer liver metastases. Ann. Gastroenterol..

[17] McQuade R.M., Stojanovska V., Bornstein J.C., Nurgali K. (2017). Colorectal Cancer Chemotherapy: The Evolution of Treatment and New Approaches. Curr. Med. Chem..

[18] Tam S.Y., Wu V.W.C. (2019). A review on the special radiotherapy techniques of colorectal cancer. Front. Oncol..

[19] Xie Y.H., Chen Y.X., Fang J.Y. (2020). Comprehensive review of targeted therapy for colorectal cancer. Signal Transduct. Target. Ther..

[20] Cappell M.S. (2008). Pathophysiology, Clinical Presentation, and Management of Colon Cancer. Gastroenterol. Clin. N. Am..

[21] ACS (2020). Colorectal Cancer Facts and Figures 2020–2022. Am. Cancer Soc..

[22] Song M., Chan A.T., Sun J. (2020). Influence of the Gut Microbiome, Diet, and Environment on Risk of Colorectal Cancer. Gastroenterology.

[23] Rubio C.A. (2017). Three pathways of colonic carcinogenesis in rats. Anticancer Res..

[24] Oh B.Y., Hong H.K., Lee W.Y., Cho Y.B. (2017). Animal models of colorectal cancer with liver metastasis. Cancer Lett..

[25] Machado V.F., Parra R.S., Leite C.A., Minto S.B., Cunha T.M., Cunha F.Q., Garcia S.B., Feitosa M.R., Da Rocha J.J.R., Feres O. (2020). Experimental model of rectal carcinogenesis induced by n-methyl-n-nitrosoguanidine in mice with endoscopic evaluation. Int. J. Med. Sci..

[26] Mittal V.K., Singh Bhullar J., Kumar J. (2015). Animal models of human colorectal cancer: Current status, uses and limitations. World J. Gastroenterol..

[27] Rosenberg D.W., Giardina C., Tanaka T. (2009). Mouse models for the study of colon carcinogenesis. Carcinogenesis.

[28] Yuan C., Zhao X., Wangmo D., Alshareef D., Gates T.J., Subramanian S. (2022). Tumor models to assess immune response and tumor-microbiome interactions in colorectal cancer. Pharmacol. Ther..

[29] Nascimento-Gonçalves E., Mendes B.A.L., Silva-Reis R., Faustino-Rocha A.I., Gama A., Oliveira P.A. (2021). Animal models of colorectal cancer: From spontaneous to genetically engineered models and their applications. Vet. Sci..

[30] Jackstadt R., Sansom O.J. (2016). Mouse models of intestinal cancer. J. Pathol..

[31] Venkatachalam K., Vinayagam R., Anand M.A.V., Isa N.M., Ponnaiyan R. (2020). Biochemical and molecular aspects of 1,2-dimethylhydrazine (DMH)-induced colon carcinogenesis: A review. Toxicol. Res..

[32] Gurley K.E., Moser R.D., Kemp C.J. (2015). Induction of colon cancer in mice with 1,2-dimethylhydrazine. Cold Spring Harb. Protoc..

[33] Meng S., Li Y., Zang X., Jiang Z., Ning H., Li J. (2020). Effect of TLR2 on the proliferation of inflammation-related colorectal cancer and sporadic colorectal cancer. Cancer Cell. Int..

[34] Lee S.H., Kwon J.E., Cho M.L. (2018). Immunological pathogenesis of inflammatory bowel disease. Intest. Res..

[35] Osawa E., Nakajima A., Fujisawa T., Kawamura Y.I., Toyama-Sorimachi N., Nakagama H., Dohi T. (2006). Predominant T helper type 2-inflammatory responses promote murine colon cancers. Int. J. Cancer.

[36] Modesto R., Estarreja J., Silva I., Rocha J., Pinto R., Mateus V. (2022). Chemically Induced Colitis-Associated Cancer Models in Rodents for Pharmacological Modulation: A Systematic Review. J. Clin. Med..

[37] Gadaleta R.M., Garcia-Irigoyen O., Moschetta A. (2017). Exploration of Inflammatory Bowel Disease in Mice: Chemically Induced Murine Models of Inflammatory Bowel Disease (IBD). Curr. Protoc. Mouse Biol..

[38] Kumar S., Agnihotri N. (2021). Piperlongumine targets NF-κB and its downstream signaling pathways to suppress tumor growth and metastatic potential in experimental colon cancer. Mol. Cell. Biochem..

[39] Fragoso M.F., Romualdo G.R., Vanderveer L.A., Franco-Barraza J., Cukierman E., Clapper M.L., Carvalho R.F., Barbisan L.F. (2018). Lyophilized açaí pulp (Euterpe oleracea Mart) attenuates colitis-associated colon carcinogenesis while its main anthocyanin has the potential to affect the motility of colon cancer cells. Food Chem. Toxicol..

[40] Megaraj V., Ding X., Fang C., Kovalchuk N., Zhu Y., Zhang Q.Y. (2014). Role of hepatic and intestinal P450 enzymes in the metabolic activation of the colon carcinogen azoxymethane in mice. Chem. Res. Toxicol..

[41] Machado V.F., Feitosa M.R., da Rocha J.J.R., Féres O. (2016). A review of experimental models in colorectal carcinogenesis. J. Coloproctol..

[42] Stastna M., Janeckova L., Hrckulak D., Kriz V., Korinek V. (2019). Human colorectal cancer from the perspective of mouse models. Genes.

[43] Li C., Lau H.C.H., Zhang X., Yu J. (2022). Mouse Models for Application in Colorectal Cancer: Understanding the Pathogenesis and Relevance to the Human Condition. Biomedicines.

[44] Kensara O.A., El-Shemi A.G., Mohamed A.M., Refaat B., Idris S., Ahmad J. (2016). Thymoquinone subdues tumor growth and potentiates the chemopreventive effect of 5-fluorouracil on the early stages of colorectal carcinogenesis in rats. Drug Des. Dev. Ther..

[45] Amos-Landgraf J.M., Kwong L.N., Kendziorski C.M., Reichelderfer M., Torrealba J., Weichert J., Haag J.D., Chen K.S., Waller J.L., Gould M.N. (2007). A target-selected Apc-mutant rat kindred enhances the modeling of familial human colon cancer. Proc. Natl. Acad. Sci. USA.

[46] Tanimura Y., Fukui T., Horitani S., Matsumoto Y., Miyamoto S., Suzuki R., Tanaka T., Tomiyama T., Ikeura T., Ando Y. (2021). Long-term model of colitis-associated colorectal cancer suggests tumor spread mechanism and nature of cancer stem cells. Oncol. Lett..

[47] Xiao Y., Dai X., Li K., Gui G., Liu J., Yang H. (2017). Clostridium butyricum partially regulates the development of colitis-associated cancer through miR-200c. Cell Mol. Biol..

[48] Clapper M.L., Cooper H.S., Chang W.C.L. (2007). Dextran sulfate sodium-induced colitis-associated neoplasia: A promising model for the development of chemopreventive interventions. Acta Pharmacol. Sin..

[49] Tong Y., Yang W., Koeffler H.P. (2011). Mouse models of colorectal cancer. Chin. J. Cancer.

[50] Evans J.P., Sutton P.A., Winiarski B.K., Fenwick S.W., Malik H.Z., Vimalachandran D., Tweedle E.M., Costello E., Palmer D.H., Park B.K. (2016). From mice to men: Murine models of colorectal cancer for use in translational research. Crit. Rev. Oncol. Hematol..

[51] National Comprehensive Cancer Network (2022). NCCN Guidelines for Patients.

[52] De Robertis M., Massi E., Poeta M.L., Carotti S., Morini S., Cecchetelli L., Signori E., Fazio V.M. (2011). The AOM/DSS murine model for the study of colon carcinogenesis: From pathways to diagnosis and therapy studies. J. Carcinog..

[53] Durmus S., van der Valk M., Teunissen S.F., Song J.Y., Wagenaar E., Beijnen J.H., Schinkel A.H. (2019). ABC transporters Mdr1a/1b, Bcrp1, Mrp2 and Mrp3 determine the sensitivity to PhIP/DSS-induced colon carcinogenesis and inflammation. Arch. Toxicol..

[54] Chen J.X., Wang H., Liu A., Zhang L., Reuhl K., Yang C.S. (2017). PhIP/DSS-induced colon carcinogenesis in CYP1A-humanized mice and the possible role of Lgr5+ stem cells. Toxicol. Sci..

[55] Wang H., Zhou H., Liu A., Guo X., Yang C.S. (2016). Genetic analysis of colon tumors induced by a dietary carcinogen PhIP in CYP1A humanized mice: Identification of mutation of β-catenin/Ctnnb1 as the driver gene for the carcinogenesis. Mol. Carcinog..

[56] Lorenz E., Stewart H.L. (1941). Intestinal carcinoma and other lesions in mice following oral administration of 1,2,5,6-dibenzanthracene and 20- methyleholanthrene. J. Natl. Cancer Inst..

[57] Walpole A.L., Williams M.H., Roberts D.C. (1952). The carcinogenic action of 4-aminodiphenyl and 3:2’-dimethyl-4-amino-diphenyl. Br. J. Ind. Med..

[58] Reddy B.S., Mori H. (1981). Effect of dietary wheat bran and dehydrated citrus fiber on 3,2′-dimethyl-4-aminobiphenyl-induced intestinal carcinogenesis in F344 rats. Carcinogenesis.

[59] Ravoori S., Feng Y., Neale J.R., Jeyabalan J., Srinivasan C., Hein D.W., Gupta R.C. (2008). Dose-dependent reduction of 3,2’-dimethyl-4-aminobiphenylderived DNA adducts in colon and liver of rats administered celecoxib. Mutat. Res..

[60] Komala M., Radhika M.N., Anbu J., Pyngrope K.R. (2019). A review on chemical models of colorectal cancer: Criteria with mechanism of carcinogenesis. Int. J. Pharm. Sci. Rev. Res..

[61] Feng Y., Fretland A.J., Rustan T.D., Jiang W., Becker W.K., Hein D.W. (1997). Higher frequency of aberrant crypt foci in rapid than slow acetylator inbred rats administered the colon carcinogen 3,2’-dimethyl-4-aminobiphenyl. Toxicol. Appl. Pharmacol..

[62] Narisawa T., Weisburger J.H. (1975). Colon cancer induction in mice by intrarectal instillation of N-methylnitosorurea (38498). Biol. Med. Proc. Soc. Exp..

[63] Narisawa T., Sato T., Hayakawa M., Sakuma A., Nakano H. (1971). Carcinoma of the colon and rectum of rats by rectal infusion of N-methyl-N’-nitro-N-nitrosoguanidine. Gann.

[64] Narisawa T., Wong C.Q., Maronpot R.R., Weisburger J.H. (1976). Large bowel carcinogenesis in mice and rats by several intrarectal doses of methylnitrosourea and negative effect of nitrite plus methylurea. Cancer Res..

[65] Qin X., Zarkovic M., Nakatsuru Y., Arai M., Oda H., Ishikawa T. (1994). DNA adduct formation and assessment of aberrant crypt foci in vivo in the rat colon mucosa after treatment with N-methyl-N-nitrosourea. Carcinogenesis.

[66] Endo T., Ookawa K., Tanaka M., Nakaji S., Tsuchida S., Sugawara K. (2001). Differences in carcinogenesis by the length of carcinogen exposure period in rat colon. Dig. Dis. Sci..

[67] Johnson R.L., Fleet J.C. (2013). Animal models of colorectal cancer. Cancer Metastasis Rev..

[68] Karakurt S., Durmus I.M., Erturk S. (2020). Handbook of Animal Models and Its Uses in Cancer Research.

[69] Alshaman R., Alattar A., El-Sayed R.M., Gardouh A.R., Elshaer R.E., Elkazaz A.Y., Eladl M.A., El-Sherbiny M., Farag N.E., Hamdan A.M. (2022). Formulation and Characterization of Doxycycline-Loaded Polymeric Nanoparticles for Testing Antitumor/Antiangiogenic Action in Experimental Colon Cancer in Mice. Nanomaterials.

[70] Shree A., Islam J., Sultana S. (2021). Quercetin ameliorates reactive oxygen species generation, inflammation, mucus depletion, goblet disintegration, and tumor multiplicity in colon cancer: Probable role of adenomatous polyposis coli, β-catenin. Phyther Res..

[71] Alkhuriji A.F., Alsaiari S.G., Alomar S.Y., Alnafjan A.A., Alobaid H., El-Khadragy M.F. (2021). Effect of mesenchymal stem cells on cytochrome-c release and inflammation in colon cancer induced by 1,2-dimethylhydrazine in Wistar albino rats. Biosci. Rep..

[72] Ertekin T., Ekinci N., Karaca O., Nisari M., Canoz O., Ulger H. (2013). Effect of angiostatin on 1,2-dimethylhydrazine-induced colon cancer in mice. Toxicol. Ind. Health.

[73] Wang Y., Jin H.-Y., Fang M.-Z., Wang X.-F., Chen H., Huang S.-L., Kong D.-S., Li M., Zhang X., Sun Y. (2020). Epigallocatechin gallate inhibits dimethylhydrazine-induced colorectal cancer in rats. World J. Gastroenterol..

[74] Eissa M.M., Ismail C.A., El-Azzouni M.Z., Ghazy A.A., Hadi M.A. (2019). Immuno-therapeutic potential of Schistosoma mansoni and Trichinella spiralis antigens in a murine model of colon cancer. Investig. New Drugs.

[75] El Joumaa M.M., Taleb R.I., Rizk S., Borjac J.M. (2020). Protective effect of matricaria chamomilla extract against 1,2-dimethylhydrazine-induced colorectal cancer in mice. J. Complement. Integr. Med..

[76] Schiller C.M., Curley W.H., McConnell E.E. (1980). Induction of colon tumors by a single oral dose of 1,2-dimethylhydrazine. Cancer Lett..

[77] Goldberg M.T., Schop R.N., Reidy J.A. (1991). Assessment of 1,2-dimethylhydrazine in bone marrow micronucleus assay: Variations in protocol and response. Environ. Mol. Mutagen..

[78] Zhu Q., Jin Z., Wu W., Gao R., Guo B., Gao Z., Yang Y., Qin H. (2014). Analysis of the intestinal lumen microbiota in an animal model of colorectal cancer. PLoS ONE.

[79] Wang C., Qiao X., Wang J., Yang J., Yang C., Qiao Y., Guan Y., Wen A., Jiang L. (2022). Amelioration of DMH-induced colon cancer by eupafolin through the reprogramming of apoptosis-associated p53/Bcl2/Bax signaling in rats. Eur. J. Inflamm..

[80] Zhao C., Ghosh B., Chakraborty T., Roy S. (2020). Bavachinin mitigates DMH induced colon cancer in rats by altering p53/Bcl2/BAX signaling associated with apoptosis. Biotech. Histochem..

[81] Babu S.S.N., Singla S., Jena G. (2022). Role of Combination Treatment of Aspirin and Zinc in DMH-DSS-induced Colon Inflammation, Oxidative Stress and Tumour Progression in Male BALB/c Mice. Biol. Trace Elem. Res..

[82] Lin P.Y., Li S.C., Lin H.P., Shih C.K. (2019). Germinated brown rice combined with Lactobacillus acidophilus and Bifidobacterium animalis subsp. lactis inhibits colorectal carcinogenesis in rats. Food Sci. Nutr..

[83] Mundo A.I., Muhammad A., Balza K., Nelson C.E., Muldoon T.J. (2022). Longitudinal examination of perfusion and angiogenesis markers in primary colorectal tumors shows distinct signatures for metronomic and maximum-tolerated dose strategies R. Neoplasia.

[84] Tian Y., Zuo L., Guan B., Wu H., He Y., Xu Z., Shen M., Hu J., Qian J. (2022). Microbiota from patients with ulcerative colitis promote colorectal carcinogenesis in mice. Nutrition.

[85] Arango-Varela S.S., Luzardo-Ocampo I., Maldonado-Celis M.E. (2022). Andean berry (Vaccinium meridionale Swartz) juice, in combination with Aspirin, displayed antiproliferative and pro-apoptotic mechanisms in vitro while exhibiting protective effects against AOM-induced colorectal cancer in vivo. Food Res. Int..

[86] Dikeocha I.J., Al-Kabsi A.M., Chiu H.T., Alshawsh M.A. (2022). Faecalibacterium prausnitzii Ameliorates Colorectal Tumorigenesis and Suppresses Proliferation of HCT116 Colorectal Cancer Cells. Biomedicines.

[87] Li Y., Zhang F., Zheng H., Kalasabail S., Hicks C., Fung K.Y., Preaudet A., Putoczki T., Beretov J., Millar EK A. (2022). Fecal DNA Virome Is Associated with the Development of Colorectal Neoplasia in a Murine Model of Colorectal Cancer. Pathogens.

[88] Almaimani R.A., Aslam A., Ahmad J., El-Readi M.Z., El-Boshy M.E., Abdelghany A.H., Idris S., Alhadrami M., Althubiti M., Almasmoum H.A. (2022). In Vivo and In Vitro Enhanced Tumoricidal Effects of of Metformin, Active Vitamin D3, and 5-Fluorouracil Triple Therapy against Colon Cancer by Modulating the PI3K/Akt/PTEN/mTOR Network. Cancers.

[89] Bähr I., Jaeschke L., Nimptsch K., Janke J., Herrmann P., Kobelt D., Kielstein H., Pischon T., Stein U. (2022). Obesity, colorectal cancer and MACC1 expression: A possible novel molecular association. Int. J. Oncol..

[90] Iwama N., Matsuda M., Tsuruta M., Okabayashi K., Shigeta K., Kanai T., Kitagawa Y. (2022). Relationship between obesity-related colorectal tumors and the intestinal microbiome: An animal-based trial. J. Cancer Res. Clin. Oncol..

[91] Ma F., Song Y., Sun M., Wang A., Jiang S., Mu G., Tuo Y. (2021). Exopolysaccharide produced by lactiplantibacillus plantarum-12 alleviates intestinal inflammation and colon cancer symptoms by modulating the gut microbiome and metabolites of C57BL/6 mice treated by azoxymethane/dextran sulfate sodium salt. Foods.

[92] Deng J., Zhao L., Yuan X., Li Y., Shi J., Zhang H., Zhao Y., Han L., Wang H., Yan Y. (2022). Pre-Administration of Berberine Exerts Chemopreventive Effects in AOM/DSS-Induced Colitis-Associated Carcinogenesis Mice via Modulating Inflammation and Intestinal Microbiota. Nutrients.

[93] Schepelmann M., Kupper N., Gushchina V., Mesteri I., Manhardt T., Moritsch S., Müller C., Piatek K., Salzmann M., Vlasaty A. (2022). AOM/DSS Induced Colitis-Associated Colorectal Cancer in 14-Month-Old Female Balb/C and C57/Bl6 Mice—A Pilot Study. Int. J. Mol. Sci..

[94] Yang M., Zhang F., Yang C., Wang L., Sung J., Garg P., Zhang M., Merlin D. (2020). Oral targeted delivery by nanoparticles enhances efficacy of an Hsp90 inhibitor by reducing systemic exposure in murine models of colitis and colitis-associated cancer. J. Crohns Colitis.

[95] Ferreira-Lazarte A., Fernández J., Gallego-Lobillo P., Villar C.J., Lombó F., Moreno F.J., Villamiel M. (2021). Behaviour of citrus pectin and modified citrus pectin in an azoxymethane/dextran sodium sulfate (AOM/DSS)-induced rat colorectal carcinogenesis model. Int. J. Biol. Macromol..

[96] Tajasuwan L., Kettawan A., Rungruang T., Wunjuntuk K., Prombutara P., Muangnoi C., Kettawan A. (2022). Inhibitory Effect of Dietary Defatted Rice Bran in an AOM/DSS-Induced Colitis-Associated Colorectal Cancer Experimental Animal Model. Foods.

[97] Bala P., Rennhack J.P., Aitymbayev D., Morris C., Moyer S.M., Duronio G.N., Doan P., LI Z., Liang X., Hornick J.L. (2023). Aberrant cell state plasticity mediated by developmental reprogramming precedes colorectal cancer initiation. Sci. Adv..

[98] Yang X., Peng H., Luo Z., Luo A., Cai M., Xu L., Wang H. (2021). The dietary carcinogen PhIP activates p53-dependent DNA damage response in the colon of CYP1A-humanized mice. BioFactors.

[99] Chen J.X., Liu A., Lee M.J., Wang H., Yu S., Chi E., Reuhl K., Suh N., Yang C. (2017). δ- and γ-tocopherols inhibit phIP/DSS-induced colon carcinogenesis by protection against early cellular and DNA damages. Mol. Carcinog..

[100] Frajacomo F.T., Kannen V., Deminice R., Geraldino T.H., Pereira-Da-Silva G., Uyemura S.A., Jordão-Jr A.A., Garcia S.B. (2015). Aerobic Training Activates Interleukin 10 for Colon Anticarcinogenic Effects. Med. Sci. Sport Exerc..

[101] Kannen V., Hintzsche H., Zanette D.L., Silva W.A., Garcia S.B., Waaga-Gasser A.M., Stopper H. (2012). Antiproliferative Effects of Fluoxetine on Colon Cancer Cells and in a Colonic Carcinogen Mouse Model. PLoS ONE.

[102] Yusuf A., Odeh O.E., Alhassan S.O., Atawodi S.E. (2022). Evaluation of the preventive potential of graded dietary inclusion of Hyphaene thebaica (Linn) fruit in rat model of colon carcinogenesis. J. Food Biochem..

[103] Qayum A., Singh J., Kumar A., Shah S.M., Srivastava S., Kushwaha M., Magotra A., Nandi U., Malik R., Sha B.A. (2022). 2-Pyridin-4-yl-methylene-beta-boswellic Acid-A Potential Candidate for Targeting O6-Methylguanine-DNA Methyltransferase Epi-transcriptional Reprogramming in KRAS G13D-Microsatellite Stable, G12V-Microsatellite Instable Mutant Colon Cancer. ACS Pharmacol. Transl. Sci..

[104] Huang Z., Liu C.A., Cai P.Z., Xu F.P., Zhu W.J., Wang W.W., Jiang H.P. (2020). Omega-3PUFA attenuates mnu-induced colorectal cancer in rats by blocking PI3K/AKT/ BCL-2 signaling. Oncol. Targets Ther..

[105] Attia N.A., Sayed A.H., Mahmoud N.S., Ahmed H.H. (2017). Phytochemical remedies: A key strategy towards reversing the aggressive murine colon cancer. Med. Chem. Res..

[106] Ahmed H.H., El-Abhar H.S., Hassanin E.A.K., Abdelkader N.F., Shalaby M.B. (2017). Punica granatum suppresses colon cancer through downregulation of Wnt/β-catenin in rat model. Rev. Bras. Farmacogn..

[107] Ahmed H.H., El-Abhar H.S., Hassanin E.A.K., Abdelkader N.F., Shalaby M.B., Ginkgo Biloba L. (2017). Leaf extract offers multiple mechanisms in bridling N-methylnitrosourea—Mediated experimental colorectal cancer. Biomed. Pharmacother..

[108] Nakayama Y., Inoue Y., Minagawa N., Onitsuka K., Nagata J., Shibao K., Hirata K., Sako T., Nagata N., Yamaguchi K. (2009). Chemopreventive effect of 4-[3,5-bis(trimethylsilyl) benzamido] benzoic acid (TAC-101) on MNU-induced colon carcinogenesis in a rat model. Anticancer Res..

[109] De-Souza A.S.C., Costa-Casagrande T.A. (2018). Animal model for colorectal cancer. ABCD Arq. Bras. Cir. Dig..

[110] Cefalì M., Epistolio S., Palmarocchi M.C., Frattini M., De Dosso S. (2021). Research progress on KRAS mutations in colorectal cancer. J. Cancer Metastasis Treat..

[111] Peehl D.M., Badea C.T., Chenevert T.L., Daldrup-Link H.E., Ding L., Dobrolecki L.E., Houghton A.M., Kinahan P.E., Kurhanewicz J., Lewis M.T. (2023). Animal Models and Their Role in Imaging-Assisted Co-Clinical Trials. Tomography.

[112] Chandra R., Karalis J.D., Liu C., Murimwa G.Z., Park J.V., Heid C.A., Resnik S.I., Huang E., Minna J.D., Brekken R.A. (2021). The Colorectal Cancer Tumor Microenvironment and Its Impact on Liver and Lung Metastasis. Cancers.

[113] Hassin O., Nataraj N.B., Shreberk-Shaked M., Aylon Y., Yaeger R., Fontemaggi G., Mukherjee S., Maddalena M., Avioz A., Iancu O. (2022). Different hotspot p53 mutants exert distinct phenotypes and predict outcome of colorectal cancer patients. Nat. Commun..

[114] Hankey W., Frankel W.L., Groden J. (2018). Functions of the APC tumor suppressor protein dependent and independent of canonical WNT signaling: Implications for therapeutic targeting. Physiol. Behav..

[115] Ren J., Sui H., Fang F., Li Q., Li B. (2019). The application of Apc Min/+ mouse model in colorectal tumor researches. J. Cancer Res. Clin. Oncol..

[116] Mcintyre R.E., Buczacki S.J.A., Arends M.J., Adams D.J. (2015). Mouse models of colorectal cancer as preclinical models. BioEssays.

[117] Bürtin F., Mullins C.S., Linnebacher M. (2020). Mouse models of colorectal cancer: Past, present and future perspectives. World J. Gastroenterol..

[118] Karim B.O., Huso D.L. (2013). Mouse models for colorectal cancer. Am. J. Cancer Res..

[119] Liu X.M., Zhu W.T., Jia M.L., Li Y.T., Hong Y., Liu Z.Q., Yan P.K. (2022). Rapamycin Liposomes Combined with 5-Fluorouracil Inhibits Angiogenesis and Tumor Growth of APC(Min/+) Mice and AOM/ DSS-Induced Colorectal Cancer Mice. Int. J. Nanomed..

[120] Metzger R., Maruskova M., Krebs S., Janssen K.P., Krug A.B. (2019). Increased Incidence of Colon Tumors in AOM-Treated Apc1638N/+ Mice Reveals Higher Frequency of Tumor Associated Neutrophils in Colon Than Small Intestine. Front. Oncol..

[121] Clark C.R., Starr T.K. (2016). Mouse models for the discovery of colorectal cancer driver genes. World J. Gastroenterol..

[122] Muller P.A.J., Caswell P.T., Doyle B., Iwanicki M.P., Tan E.H., Karim S., Lukashchuk N., Gillespie D.A., Ludwig R.L., Gosselin P. (2009). Mutant p53 Drives Invasion by Promoting Integrin Recycling. Cell..

[123] Sei S., Ahadova A., Keskin D.B., Bohaumilitzky L., Gebert J., von Knebel Doeberitz M., Lipkin S.M., Kloor M. (2023). Lynch syndrome cancer vaccines: A roadmap for the development of precision immunoprevention strategies. Front. Oncol..

[124] Telang N. (2021). Isolation and characterization of chemo-resistant stem cells from a mouse model of hereditary non-polyposis colon cancer. Stem Cells Cloning Adv. Appl..

[125] Pećina-Šlaus N., Kafka A., Salamon I., Bukovac A. (2020). Mismatch Repair Pathway, Genome Stability and Cancer. Front. Mol. Biosci..

[126] Valle L., Vilar E., Tavtigian S.V., Stoffel E.M. (2019). Genetic predisposition to colorectal cancer: Syndromes, genes, classification of genetic variants and implications for precision medicine. J. Pathol..

[127] Hryhorowicz S., Kaczmarek-Ryś M., Lis-Tanaś E., Porowski J., Szuman M., Grot N., Kryszczyńska A., Paszkowski J., Banasiewicz T., Pławski A. (2022). Strong Hereditary Predispositions to Colorectal Cancer. Genes.

[128] Taketo M.M., Edelmann W. (2009). Mouse Models of Colon Cancer. Gastroenterology.

[129] Heijstek M.W., Kranenburg O., Rinkes I.H.M.B. (2005). Mouse Models of Colorectal Cancer and Liver Metastases. Dig. Surg..

[130] Uronis J.M., Threadgill D.W. (2009). Murine models of colorectal cancer. Mamm. Genome.

[131] Schnell U., Cirulli V., Giepmans B.N.G. (2013). Biochimica et Biophysica Acta EpCAM: Structure and function in health and disease. BBA Biomembr..

[132] Gires O. (2020). Expression and function of epithelial cell adhesion molecule EpCAM: Where are we after 40 years?. Cancer Metastasis Rev..

[133] Hosono H., Ohishi T., Takei J., Asano T., Sayama Y., Kawada M., Kaneko M., Kato Y. (2020). The anti-epithelial cell adhesion molecule (EpCAM) monoclonal antibody EpMab-16 exerts antitumor activity in a mouse model of colorectal adenocarcinoma. Oncol. Lett..

[134] Alamo P., Gallardo A., Di Nicolantonio F., Pavón M.A., Casanova I., Trias M., Mangues M.A., Lopez-Pousa A., Villaverde A., Vázquez E. (2015). Higher metastatic efficiency of KRas G12V than KRas G13D in a colorectal cancer model. FASEB J..

[135] Biswas K., Couillard M., Cavallone L., Burkett S., Stauffer S., Martin B.K., Southon E., Reid S., Plona T.M., Baugher R.N. (2021). A novel mouse model of PMS2 founder mutation that causes mismatch repair defect due to aberrant splicing. Cell Death Dis..

[136] Oliveira R.C., Abrantes A.M., Tralhão J.G., Botelho M.F. (2020). The role of mouse models in colorectal cancer research—The need and the importance of the orthotopic models. Anim. Model. Exp. Med..

[137] Shibata H., Toyama K., Shioya H., Ito M., Hirota M., Hasegawa S., Matsumoto H., Takano H., Akiyama T., Toyoshima K. (1997). Rapid Colorectal Adenoma Formation Initiated by Conditional Targeting of the Apc Gene. Science.

[138] Xue Y., Johnson R., DeSmet M., Snyder P.W., Fleet J.C. (2010). Generation of a Transgenic Mouse for Colorectal Cancer Research with Intestinal Cre-Expression Limited to the Large Intestine. Mol. Cancer Res..

[139] Halberg R.B., Katzung D.S., Hoff P.D., Moser A.R., Cole C.E., Lubet R.A., Donehower L.A., Jacoby R.F., Dove W.F. (2000). Tumorigenesis in the multiple intestinal neoplasia mouse: Redundancy of negative regulators and specificity of modifiers. Proc. Natl. Acad. Sci. USA.

[140] Sakamoto K., Lin B., Nunomura K., Izawa T., Nakagawa S. (2022). The K-Ras(G12D)-inhibitory peptide KS-58 suppresses growth of murine CT26 colorectal cancer cell-derived tumors. Sci. Rep..

[141] Yueh A.E., Payne S.N., Leystra A.A., Van De Hey D.R., Foley T.M., Pasch C.A., Clipson L., Matkowskyj K.A., Deming D.A. (2016). Colon cancer tumorigenesis initiated by the H1047R mutant PI3K. PLoS ONE.

[142] Herberg M., Siebert S., Quaas M., Thalheim T., Rother K., Hussong M., Altmüller J., Kerner C., Galle J., Aust G. (2019). Loss of Msh2 and a single-radiation hit induce common, genome-wide, and persistent epigenetic changes in the intestine. Clin. Epigenet..

[143] Choi S.H., Huang A.Y., Letterio J.J., Kim B.G. (2022). Smad4-deficient T cells promote colitis-associated colon cancer via an IFN-γ-dependent suppression of 15-hydroxyprostaglandin dehydrogenase. Front. Immunol..

[144] Hung K.E., Maricevich M.A., Richard L.G., Chen W.Y., Richardson M.P., Kunin A., Bronson R.T., Mahmood U., Kucherlapati R. (2010). Development of a mouse model for sporadic and metastatic colon tumors and its use in assessing drug treatment. Proc. Natl. Acad. Sci. USA..

[145] Bennecke M., Kriegl L., Bajbouj M., Retzlaff K., Robine S., Jung A., Arkan M.C., Kirchner T., Greten F.R. (2010). Ink4a/Arf and oncogene-induced senescence prevent tumor progression during alternative colorectal tumorigenesis. Cancer Cell.

[146] Lo Y.H., Noah T.K., Chen M.S., Zou W., Borras E., Vilar E., Shroyer N.F. (2017). SPDEF Induces Quiescence of Colorectal Cancer Cells by Changing the Transcriptional Targets of β-catenin. Gastroenterology.

[147] Steffensen I.L., Alexander J. (2006). Impact of genetic background on spontaneous or 2-amino-1-methyl-6-phenylimidazo[4,5-b]pyridine (PhIP)-induced intestinal tumorigenesis in Min/+ mice. Cancer Lett..

[148] Oshima H., Nakayama M., Han T.S., Naoi K., Ju X., Maeda Y., Robine S., Tsuchiya K., Sato T., Sato H. (2015). Suppressing TGFβ signaling in regenerating epithelia in an inflammatory microenvironment is sufficient to cause invasive intestinal cancer. Cancer Res..

[149] Sakai E., Nakayama M., Oshima H., Kouyama Y., Niida A., Fujii S., Ochiai A., Nakayama K.I., Mimori K., Suzuki Y. (2018). Combined mutation of Apc, Kras, and Tgfbr2 effectively drives metastasis of intestinal cancer. Cancer Res..

[150] Takaku K., Oshima M., Miyoshi H., Matsui M., Seldin M.F., Taketo M.M. (1998). Intestinal tumorigenesis in compound mutant mice of both Dpc4 (Smad4) and Apc genes. Cell.

[151] Kucherlapati M., Yang K., Kuraguchi M., Zhao J., Lia M., Heyer J., Kane M.F., Fan K., Russell R., Brown A.M.C. (2002). Haploinsufficiency of Flap endonuclease (Fen1) leads to rapid tumor progression. Proc. Natl. Acad. Sci. USA.

[152] Grim J.E., Knoblaugh S.E., Guthrie K.A., Hagar A., Swanger J., Hespelt J., Delrow J.J., Small T., Grady W.M., Nakayama K.I. (2012). Fbw7 and p53 Cooperatively Suppress Advanced and Chromosomally Unstable Intestinal Cancer. Mol. Cell Biol..

[153] Sansom O.J., Meniel V., Wilkins J.A., Cole A.M., Oien K.A., Marsh V., Jamieson T.J., Guerra C., Ashton G.H., Barbacid M. (2006). Loss of Apc allows phenotypic manifestation of the transforming properties of an endogenous K-ras oncogene in vivo. Proc. Natl. Acad. Sci. USA.

[154] Tong K., Kothari O.A., Haro K.S., Panda A., Bandari M.M., Carrick J.N., Hur J.J., Zhang L., Chan C.S., Xing J. (2021). SMAD4 is critical in suppression of BRAF-V600E serrated tumorigenesis. Oncogene.

[155] Zhou H., Liu Z., Wang Y., Wen X., Amador E.H., Yuan L., Ran X., Xiong L., Ran Y., Chen W. (2022). Colorectal liver metastasis: Molecular mechanism and interventional therapy. Signal Transduct. Target. Ther. Vol..

[156] Abdolahi S., Ghazvinian Z., Muhammadnejad S., Saleh M., Asadzadeh Aghdaei H., Baghaei K. (2022). Patient-derived xenograft (PDX) models, applications and challenges in cancer research. J. Transl. Med..

[157] Talmadge J.E., Singh R.K., Fidler I.J., Raz A. (2007). Murine models to evaluate novel and conventional therapeutic strategies for cancer. Am. J. Pathol..

[158] Liu X., Xin Z., Wang K. (2022). Patient-derived xenograft model in colorectal cancer basic and translational research. Anim. Model. Exp. Med..

[159] Chen J., Liao S., Xiao Z., Pan Q., Wang X., Shen K., Wang S., Yang L., Guo F., Liu H. (2022). The development and improvement of immunodeficient mice and humanized immune system mouse models. Front. Immunol..

[160] Golovko D., Kedrin D., Yilmaz O.H., Roper J. (2015). Review: US Spelling Colorectal cancer models for novel drug discovery. Expert Opin. Drug. Discov..

[161] Medico E., Russo M., Picco G., Cancelliere C., Valtorta E., Corti G., Buscarino M., Isella C., Lamba S., Martinoglio B. (2015). The molecular landscape of colorectal cancer cell lines unveils clinically actionable kinase targets. Nat. Commun..

[162] Ben-David U., Siranosian B., Ha G., Tang H., Oren Y., Hinohara K., Strathdee C.A., Dempster J., Lyons N.J., Burns B.R. (2018). Genetic and transcriptional evolution alters cancer cell line drug response. Nature.

[163] Greenlee J.D., King M.R. (2022). A syngeneic MC38 orthotopic mouse model of colorectal cancer metastasis. Biol. Methods Protoc..

[164] Kishimoto H., Momiyama M., Aki R., Kimura H., Suetsugu A., Bouvet M., Fujiwara T., Hoffman R.M. (2013). Development of a clinically-precise mouse model of rectal cancer. PLoS ONE.

[165] Gulbake A., Jain A., Jain A., Jain A., Jain S.K. (2016). Insight to drug delivery aspects for colorectal cancer. World J. Gastroenterol..

[166] Dinger T.F., Chen O., Dittfeld C., Hetze L., Hüther M., Wondrak M., Löck S., Eicheler W., Breier G., Kunz-Schughart L.A. (2020). Microenvironmentally-driven plasticity of CD44 isoform expression determines engraftment and stem-like phenotype in CRC cell lines. Theranostics.

[167] Almajali B., Al-Jamal H.A.N., Taib W.R.W., Ismail I., Johan M.F., Doolaanea A.A., Ibrahim W.N. (2021). Thymoquinone, as a novel therapeutic candidate of cancers. Pharmaceuticals.

[168] Choi J.R., Kozalak G., Di Bari I., Babar Q., Niknam Z., Rasmi Y., Yong K.W. (2022). In Vitro Human Cancer Models for Biomedical Applications. Cancers.

[169] Rajput A., Agarwal E., Leiphrakpam P., Brattain M.G., Chowdhury S. (2013). Establishment and Validation of an Orthotopic Metastatic Mouse Model of Colorectal Cancer. ISRN Hepatol..

[170] Idrisova K.F., Simon H.U., Gomzikova M.O. (2023). Role of Patient-Derived Models of Cancer in Translational Oncology. Cancers.

[171] Hon K.W., Zainal Abidin S.A., Othman I., Naidu R. (2021). The Crosstalk Between Signaling Pathways and Cancer Metabolism in Colorectal Cancer. Front. Pharmacol..

[172] Zhang B., Liu Q., Wen W., Gao H., Wei W., Tang A., Qin B., Lyu H., Meng X., Li K. (2022). The chromatin remodeler CHD6 promotes colorectal cancer development by regulating TMEM65-mediated mitochondrial dynamics via EGF and Wnt signaling. Cell Discov..

[173] Khan F.A., Albalawi R., Pottoo F.H. (2022). Trends in targeted delivery of nanomaterials in colon cancer diagnosis and treatment. Med. Res. Rev..

[174] Páez-Franco J.C., Zermeño-Ortega M.R., de la O-Contreras C.M., Canseco-González D., Parra-Unda J.R., Avila-Sorrosa A., Enríquez R.G., Germán-Acacio J.M., Morales-Morales D. (2022). Relevance of Fluorinated Ligands to the Design of Metallodrugs for Their Potential Use in Cancer Treatment. Pharmaceutics.

[175] Dsouza V.L., Kuthethur R., Kabekkodu S.P., Chakrabarty S. (2022). Organ-on-Chip platforms to study tumor evolution and chemosensitivity. Biochim. Biophys. Acta Rev. Cancer.

[176] Klose J., Trefz S., Wagner T., Steffen L., Charrier A.P., Radhakrishnan P., Volz C., Schmidt T., Ulrich A., Dieter S.M. (2019). Salinomycin: Anti-tumor activity in a preclinical colorectal cancer model. PLoS ONE.

[177] Xu C., Li X., Liu P., Li M., Luo F. (2019). Patient-derived xenograft mouse models: A high fidelity tool for individualized medicine (review). Oncol. Lett..

[178] Calcagno S.R., Li S., Colon M., Kreinest P.A., Thompson E.A., Fields A.P., Murray N.R. (2008). Oncogenic K-ras promotes early carcinogenesis in the mouse proximal colon. Int. J. Cancer.

[179] Cook D.R., Kang M., Martin T.D., Galanko J.A., Loeza G.H., Trembath D.G., Justilien V., Pickering K.A., Vincent D.F., Jarosch A. (2021). Aberrant Expression and Subcellular Localization of ECT2 Drives Colorectal Cancer Progression and Growth. Cancer Res..

[180] Liu K., Huang L., Qi S., Liu S., Xie W., Du L., Cui J., Zhang X., Zhang B., Liu L. (2023). Ferroptosis: The Entanglement between Traditional Drugs and Nanodrugs in Tumor Therapy. Adv. Healthc. Mater..

[181] Wani W.A., Prashar S., Shreaz S., Gomez-Ruiz S. (2016). Nanostructured materials functionalized with metal complexes: In search of alternatives for administering anticancer metallodrugs. Coord. Chem. Rev..

[182] Wu S., Wang J., Fu Z., Familiari G., Relucenti M., Aschner M., Li X., Chen H., Chen R. (2023). Matairesinol Nanoparticles Restore Chemosensitivity and Suppress Colorectal Cancer Progression in Preclinical Models: Role of Lipid Metabolism Reprogramming. Nano Lett..

[183] Sun X., Ng T.T.H., Sham K.W.Y., Zhang L., Chan M.T.V., Wu W.K.K., Cheng C.H.K. (2019). Bufalin, a traditional Chinese medicine compound, prevents tumor formation in two murine models of colorectal cancer. Cancer Prev. Res..

[184] Xu Y., Zhang L., Wang Q., Zheng M. (2020). Comparison of Different Colorectal Cancer With Liver Metastases Models Using Six Colorectal Cancer Cell Lines. Pathol. Oncol. Res..

[185] Kim H.D., Park E.J., Choi E.K., Song S.Y., Hoe K.L., Kim D.U. (2021). *G*-749 Promotes Receptor Tyrosine Kinase TYRO3 Degradation and Induces Apoptosis in Both Colon Cancer Cell Lines and Xenograft Mouse Models. Front. Pharmacol..

[186] Salama A.A.A., Allam R.M. (2021). Promising targets of chrysin and daidzein in colorectal cancer: Amphiregulin, CXCL1, and MMP-9. Eur. J. Pharmacol..

[187] Teng S., Li Y.E., Yang M., Qi R., Huang Y., Wang Q., Zhang Y., Chen S., Li S., Lin K. (2020). Tissue-specific transcription reprogramming promotes liver metastasis of colorectal cancer. Cell Res..

[188] Shimura T., Toden S., Komarova N.L., Boland C.R., Wodarz D., Goel A. (2020). A comprehensive in vivo and mathematic modelingbased kinetic characterization for aspirin-induced chemoprevention in colorectal cancer. Carcinogenesis.

[189] Li C., Wang Y., Liu D., Wong C.C., Coker O.O., Zhang X., Liu C., Zhou Y., Liu Y., Kang W. (2022). Squalene epoxidase drives cancer cell proliferation and promotes gut dysbiosis to accelerate colorectal carcinogenesis. Gut.

[190] Hare J.I., Neijzen R.W., Anantha M., Dos Santos N., Harasym N., Webb M.S., Allen T.M., Bally M.B., Waterhouse D.N. (2013). Treatment of Colorectal Cancer Using a Combination of Liposomal Irinotecan (Irinophore C^TM^) and 5-Fluorouracil. PLoS ONE.

[191] Takahashi T., Morotomi M., Nomoto K. (2004). A novel mouse model of rectal cancer established by orthotopic implantation of colon cancer cells. Cancer Sci..

[192] Hollandsworth H.M., Amirfakhri S., Filemoni F., Hoffman R.M., Molnar J., Yazaki P.J., Bouvet M. (2020). Humanized Anti–Tumor-Associated Glycoprotein–72 for Submillimeter Near-Infrared Detection of Colon Cancer in Metastatic Mouse Models. J. Surg. Res..

[193] Hollandsworth H.M., Amirfakhri S., Filemoni F., Molnar J., Hoffman R.M., Yazaki P., Bouvet M. (2020). Near-infrared photoimmunotherapy is effective treatment for colorectal cancer in orthotopic nude-mouse models. PLoS ONE.

[194] Thangaiyan R., Aljahdali I.A.M., Lent-Moore K.Y., Liao J., Ling X., Li F. (2021). Kras mutation subtypes distinctly affect colorectal cancer cell sensitivity to FL118, a novel inhibitor of survivin, Mcl-1, XIAP, cIAP2 and MdmX. Am. J. Transl. Res..

[195] Murdocca M., Capuano R., Pucci S., Cicconi R., Polidoro C., Catini A., Martinelli E., Paolesse R., Orlandi A., Mango R. (2019). Targeting LOX-1 inhibits colorectal cancer metastasis in an animal model. Front. Oncol..

[196] Ko E.J., Ock M.S., Choi Y.H., Iovanna J.L., Mun S., Han K., Kim H., Cha H. (2021). Human Endogenous Retrovirus (HERV)-K env Gene Knockout Affects Tumorigenic Characteristics of nupr1 Gene in DLD-1 Colorectal Cancer Cells. Int. J. Mol. Sci..

[197] Céspedes M.V., Espina C., García-Cabezas M.A., Trias M., Boluda A., Gómez Del Pulgar M.T., Sancho F.J., Nistal M., Lacal J.C., Mangues R. (2007). Orthotopic Microinjection of Human Colon Cancer Cells in Nude Mice Induces Tumor Foci in All Clinically Relevant Metastatic Sites. Am. J. Pathol..

[198] Rivera M., Fichtner I., Wulf-Goldenberg A., Sers C., Merk J., Patone G., Alp K.M., Kanashova T., Mertins P., Hoffmann J. (2021). Patient-derived xenograft (PDX) models of colorectal carcinoma (CRC) as a platform for chemosensitivity and biomarker analysis in personalized medicine. Neoplasia US.

[199] Relucenti M., Francescangeli F., De Angelis M.L., D’Andrea V., Miglietta S., Donfrancesco O., Li X., Chen R., Zeuner A., Familiari G. (2022). A Different Exosome Secretion Pattern Characterizes Patient-Derived Colorectal Cancer Multicellular Spheroids and Their Mouse Xenografts. Biology.

[200] Hua L., Chen L., Huang J., Chen X., Guo S., Wang J. (2022). Establishment of RET inhibitor-induced resistant patient-derived colorectal cancer xenograft models. Eur. J. Cancer.

[201] Drury J., Young L.E.A., Scott T.L., Kelson C.O., He D., Liu J., Wu Y., Wang C., Weiss H.L., Fan T. (2022). Tissue-Specific Downregulation of Fatty Acid Synthase Suppresses Intestinal Adenoma Formation via Coordinated Reprograming of Transcriptome and Metabolism in the Mouse Model of Apc-Driven Colorectal Cancer. Int. J. Mol. Sci..

[202] Liu Y., Wu W., Cai C., Zhang H., Shen H., Han Y. (2023). Patient-derived xenograft models in cancer therapy: Technologies and applications. Signal. Transduct. Target. Ther..

[203] Janakiraman H., Zhu Y., Becker S.A., Wang C., Cross A., Curl E., Lewin D., Hoffman B.J., Warren G.W., Hill E.G. (2020). Modeling rectal cancer to advance neoadjuvant precision therapy. Int. J. Cancer.

[204] De Angelis M.L., Francescangeli F., Nicolazzo C., Xhelili E., La Torre F., Colace L., Bruselles A., Macchia D., Vitale S., Gazzaniga P. (2022). An Orthotopic Patient-Derived Xenograft (PDX) Model Allows the Analysis of Metastasis-Associated Features in Colorectal Cancer. Front. Oncol..

[205] Cassidy J.W., Caldas C., Bruna A. (2015). Maintaining tumor heterogeneity in patient-derived tumor xenografts. Cancer Res..

[206] Cho S.-Y. (2020). Patient-derived xenografts as compatible models for precision oncology. Lab. Anim. Res..

[207] Ramzy G.M., Koessler T., Ducrey E., McKee T., Ris F., Buchs N., Rubbia-Brandt L., Dietrich P.-Y., Nowak-Sliwinska P. (2020). Patient-derived in vitro models for drug discovery in colorectal carcinoma. Cancers.

[208] Yamaguchi N., Weinberg E.M., Nguyen A., Liberti M.V., Goodarzi H., Janjigian Y.Y., Paty P.B., Saltz L.B., Kingham T.P., Loo J.M. (2019). PCK1 and DHODH drive colorectal cancer liver metastatic colonization and hypoxic growth by promoting nucleotide synthesis. Elife.

[209] Chen C., Lin W., Huang Y., Chen X., Wang H., Teng L. (2021). The essential factors of establishing patient-derived tumor model. J. Cancer.

[210] Wang E., Xiang K., Zhang Y., Wang X.F. (2022). Patient-derived organoids (PDOs) and PDO-derived xenografts (PDOXs): New opportunities in establishing faithful pre-clinical cancer models. J. Natl. Cancer Cent..

[211] Hassani I., Anbiah B., Kuhlers P., Habbit N.L., Ahmed B., Heslin M.J., Mobley J.A., Greene M.W., Lipke E.A. (2022). Engineered colorectal cancer tissue recapitulates key attributes of a patient-derived xenograft tumor line. Biofabrication.

[212] Fondevila F., Méndez-Blanco C., Fernández-Palanca P., González-Gallego J., Mauriz J.L. (2019). Anti-tumoral activity of single and combined regorafenib treatments in preclinical models of liver and gastrointestinal cancers. Exp. Mol. Med..

[213] Vaghi C., Mauri G., Giuseppe Agostara A., Patelli G., Gregory Pizzutilo E., Nakamura Y., Yoshino T., Siena S., Sartore-Bianchi A. (2022). The predictive role of ERBB2 point mutations in metastatic colorectal cancer: A systematic review. Cancer Treat. Rev..

[214] La Salvia A., Lopez-Gomez V., Garcia-Carbonero R. (2019). HER2-targeted therapy: An emerging strategy in advanced colorectal cancer. Expert. Opin. Investig. Drugs.

[215] Lazzari L., Corti G., Picco G., Isella C., Montone M., Arcela P., Durinikova E., Zanella E.R., Novara L., Barbosa F. (2019). Patient-Derived Xenografts and Matched Cell Lines Identify Pharmacogenomic Vulnerabilities in Colorectal Cancer. Clin. Cancer Res..

[216] Wagner S., Beger N.T., Matschos S., Szymanski A., Przybylla R., Bürtin F., Prall F., Linnebacher M., Mullins C.S. (2021). Tumour-Derived Cell Lines and Their Potential for Therapy Prediction in Patients with Metastatic Colorectal Cancer. Cancers.

[217] Verginelli F., Pisacane A., Gambardella G., D’Ambrosio A., Candiello E., Ferrio M., Panero M., Casorzo L., Benvenuti S., Cascardi E. (2021). Cancer of unknown primary stem-like cells model multi-organ metastasis and unveil liability to MEK inhibition. Nat. Commun..

[218] Madan B., Harmston N., Nallan G., Montoya A., Faull P., Petretto E., Virshup D.M. (2018). Temporal dynamics of Wnt-dependent transcriptome reveal an oncogenic Wnt/MYC/ribosome axis. J. Clin. Investig..

[219] Hou X., Du C., Lu L., Yuan S., Zhan M., You P., Du H. (2022). Opportunities and challenges of patient-derived models in cancer research: Patient-derived xenografts, patient-derived organoid and patient-derived cells. World J. Surg. Oncol..

[220] Ivanics T., Bergquist J.R., Liu G., Kim M.P., Kang Y., Katz M.H., Perez M.V.R., Thomas R.M., Truty M.J. (2018). Patient-derived xenograft cryopreservation and reanimation outcomes are dependent on cryoprotectant type. Lab. Investig..

[221] Pyo D.H., Hong H.K., Lee W.Y., Cho Y.B. (2020). Patient-derived cancer modeling for precision medicine in colorectal cancer: Beyond the cancer cell line. Cancer Biol. Ther..

[222] Kanikarla Marie P., Sorokin A.V., Bitner L.A., Aden R., Lam M., Manyam G., Woods M.N., Anderson A., Capasso A., Fowlkes N. (2022). Autologous humanized mouse models to study combination and single-agent immunotherapy for colorectal cancer patient-derived xenografts. Front. Oncol..

[223] Choi Y., Lee S., Kim K., Kim S.H., Chung Y.J., Lee C. (2018). Studying cancer immunotherapy using patient-derived xenografts (PDXs) in humanized mice. Exp. Mol. Med..

[224] Kwisda K., White L., Hübner D. (2020). Ethical arguments concerning human-animal chimera research: A systematic review. BMC Med. Ethics.

[225] Inoue A., Deem A.K., Kopetz S., Heffernan T.P., Draetta G.F., Carugo A. (2019). Current and future horizons of patient-derived xenograft models in colorectal cancer translational research. Cancers.

[226] Shi J., Li Y., Jia R., Fan X. (2020). The fidelity of cancer cells in PDX models: Characteristics, mechanism and clinical significance. Int. J. Cancer.

[227] Lee A.Q., Ijiri M., Rodriguez R., Gandour-Edwards R., Lee J., Tepper C.G., Li Y., Beckett L., Lam K., Goodwin N. (2021). Novel Patient Metastatic Pleural Effusion-Derived Xenograft Model of Renal Medullary Carcinoma Demonstrates Therapeutic Efficacy of Sunitinib. Front. Oncol..

[228] Tayoun T., Faugeroux V., Oulhen M., Aberlenc A., Pawlikowska P., Farace F. (2019). CTC-Derived Models: A Window into the Seeding Capacity of Circulating Tumor Cells (CTCs). Cells.

[229] Yang R., Yu Y. (2023). Patient-derived organoids in translational oncology and drug screening. Cancer Lett..

[230] Zhou Z., Cong L., Cong X. (2021). Patient-Derived Organoids in Precision Medicine: Drug Screening, Organoid-on-a-Chip and Living Organoid Biobank. Front. Oncol..

[231] Bae J., Choi Y.S., Cho G., Jang S.J. (2022). The Patient-Derived Cancer Organoids: Promises and Challenges as Platforms for Cancer Discovery. Cancers.

[232] Liu Y.C., Chen Q., Yang X.L., Tang Q.S., Yao K.T., Xu Y. (2018). Generation of a new strain of NOD/SCID/IL2Rγ-/- mice with targeted disruption of Prkdc and IL2Rγ genes using CRISPR/Cas9 system. Nan Fang Yi Ke Da Xue Xue Bao.

[233] Zhang H., Qi L., Du Y., Huang L.F., Braun F.K., Kogiso M., Zhao Y., Li C., Lindsay H., Zhao S. (2020). Patient-derived orthotopic xenograft (PDOX) mouse models of primary and recurrent meningioma. Cancers.

[234] Liu C., Wang T., Yang J., Zhang J., Wei S., Guo Y., Yu R., Tan Z., Wang S., Dong W. (2022). Distant Metastasis Pattern and Prognostic Prediction Model of Colorectal Cancer Patients Based on Big Data Mining. Front. Oncol..

[235] Hsu P.-L., Chien C.-W., Tang Y.-A., Lin B.-W., Lin S.-C., Lin Y.-S., Chen S.-Y., Sun H.S., Tsai S.-J. (2023). Targeting BRD3 eradicates nuclear TYRO3-induced colorectal cancer metastasis. Sci. Adv..

[236] Jian M., Ren L., Ren L., He G., He G., Lin Q., Chen J., Liu T., Ji M., Wei Y. (2020). A novel patient-derived organoids-based xenografts model for preclinical drug response testing in patients with colorectal liver metastases. J. Transl. Med..

[237] Andres S.F., Williams K.N., Rustgi A.K. (2018). The Molecular Basis of Metastatic Colorectal Cancer. Curr. Color. Cancer Rep..

[238] Guo J., Yu Z., Das M., Huang L. (2020). Nano Codelivery of Oxaliplatin and Folinic Acid Achieves Synergistic Chemo-Immunotherapy with 5-Fluorouracil for Colorectal Cancer and Liver Metastasis. ACS Nano.

[239] Kasashima H., Duran A., Cid-Diaz T., Muta Y., Kinoshita H., Batlle E., Diaz-Meco M.T., Moscat J. (2021). Mouse model of colorectal cancer: Orthotopic co-implantation of tumor and stroma cells in cecum and rectum. STAR Protoc..

[240] Rashidi B., Gamagami R., Sasson A., Sun F.X., Geller J., Moossa A.R., Hoffman R.M. (2000). An orthotopic mouse model of remetastasis of human colon cancer liver metastasis. Clin. Cancer Res..

[241] Schwitalla S., Ziegler P.K., Horst D., Becker V., Kerle I., Begus-Nahrmann Y., Lechel A., Rudolph K.L., Langer R., Slotta-Huspenina J. (2013). Loss of p53 in Enterocytes Generates an Inflammatory Microenvironment Enabling Invasion and Lymph Node Metastasis of Carcinogen-Induced Colorectal Tumors. Cancer Cell.

[242] Dow L.E., O’Rourke K.P., Simon J., Tschaharganeh D.F., Van Es J.H., Clevers H., Lowe S.W. (2015). Apc restoration promotes cellular differentiation and reestablishes crypt homeostasis in colorectal cancer. Cell.

[243] Boutin A.T., Liao W.T., Wang M., Hwang S.S., Karpinets T.V., Cheung H., Chu G.C., Jiang S., Hu J., Chang K. (2017). Oncogenic Kras drives invasion and maintains metastases in colorectal cancer. Genes Dev..

[244] Rampetsreiter P., Casanova E., Eferl R. (2011). Genetically modified mouse models of cancer invasion and metastasis. Drug. Discov. Today Dis. Model..

[245] Takasago T., Hayashi R., Ueno Y., Ariyoshi M., Onishi K., Yamashita K., Hiyama Y., Takigawa H., Yuge R., Urabe Y. (2023). Anti-tumor necrosis factor-alpha monoclonal antibody suppresses colorectal cancer growth in an orthotopic transplant mouse model. PLoS ONE.

[246] Zhang Q., Yang X., Wu J., Ye S., Gong J., Cheng W.M., Luo Z., Yu J., Liu Y., Zeng W. (2023). Reprogramming of palmitic acid induced by dephosphorylation of ACOX1 promotes β-catenin palmitoylation to drive colorectal cancer progression. Cell. Discov..

[247] Zeng X., Yao B., Liu J., Gong G.-W., Liu M., Li J., Pan H.-F., Li Q., Yang D., Lu P. (2023). The SMARCA4R1157W mutation facilitates chromatin remodeling and confers PRMT1/SMARCA4 inhibitors sensitivity in colorectal cancer. npj Precis. Oncol..

[248] Xu H., Luo H., Zhang J., Li K., Lee M.H. (2023). Therapeutic potential of Clostridium butyricum anticancer effects in colorectal cancer. Gut Microbes.

[249] Lattanzi G., Strati F., Díaz-Basabe A., Perillo F., Amoroso C., Protti G., Rita Giuffrè M., Iachini L., Baeri A., Baldari L. (2023). iNKT cell-neutrophil crosstalk promotes colorectal cancer pathogenesis. Mucosal Immunol..

[250] Cave D.D., Hernando-Momblona X., Sevillano M., Minchiotti G., Lonardo E. (2021). Nodal-induced L1CAM/CXCR4 subpopulation sustains tumor growth and metastasis in colorectal cancer derived organoids. Theranostics.

[251] Zhang Y., Lee S.H., Wang C., Gao Y., Li J., Xu W. (2020). Establishing metastatic patient-derived xenograft model for colorectal cancer. Jpn. J. Clin. Oncol..

[252] Yang H., Li X., Meng Q., Sun H., Wu S., Hu W., Liu G., Li X., Yang Y., Chen R. (2020). CircPTK2 (hsa_circ_0005273) as a novel therapeutic target for metastatic colorectal cancer. Mol. Cancer.

[253] Torchiaro E., Petti C., Arena S., Sassi F., Migliardi G., Mellano A., Porporato R., Basiricò M., Gammaitoni L., Berrino E. (2023). Case report: Preclinical efficacy of NEDD8 and proteasome inhibitors in patient-derived models of signet ring high-grade mucinous colorectal cancer from a Lynch syndrome patient. Front. Oncol..

[254] Sun H., Liu C., Han F., Lin X., Cao L., Liu C., Ji Q., Cui J., Yao Y., Wang B. (2023). The regulation loop of MARVELD1 interacting with PARP1 in DNA damage response maintains genome stability and promotes therapy resistance of cancer cells. Cell Death Differ..

[255] Shang Y., Zhu Z., Zhang Y., Ji F., Zhu L., Liu M., Deng Y., Lv G., Li D., Zhou Z. (2023). MiR-7-5p/KLF4 signaling inhibits stemness and radioresistance in colorectal cancer. Cell Death Discov..

[256] Fujii M., Shimokawa M., Date S., Takano A., Matano M., Nanki K., Ohta Y., Toshimitsu K., Nakazato Y., Kawasaki K. (2016). A Colorectal Tumor Organoid Library Demonstrates Progressive Loss of Niche Factor Requirements during Tumorigenesis. Cell Stem Cell.

[257] Vlachogiannis G., Hedayat S., Vatsiou A., Jamin Y., Fernández-mateos J., Khan K., Lampis A., Eason K., Huntingford I., Burke R. (2018). Patient-derived organoids model treatment response of metastatic gastrointestinal cancers. Science.

[258] Kersten K., Visser K.E., Miltenburg M.H., Jonkers J. (2017). Genetically engineered mouse models in oncology research and cancer medicine. EMBO Mol. Med..

[259] Avolio M., Trusolino L. (2021). Rational treatment of metastatic colorectal cancer: A reverse tale of men, mice, and culture dishes. Cancer Discov..

[260] Kwon J., Oh S., Park M., Kong J.S., Lee S., Lee H., Kim Y., Kang K.-T., Shin U.S., Jung J. (2021). Advanced Xenograft Model with Cotransplantation of Patient-Derived Organoids and Endothelial Colony-Forming Cells for Precision Medicine. J. Oncol..

[261] Kang K.T., Lin R.Z., Kuppermann D., Melero-Martin J.M., Bischoff J. (2017). Endothelial colony forming cells and mesenchymal progenitor cells form blood vessels and increase blood flow in ischemic muscle. Sci. Rep..

[262] Matano M., Date S., Shimokawa M., Takano A., Fujii M., Ohta Y., Watanabe T., Kanai T., Sato T. (2015). Modeling colorectal cancer using CRISPR-Cas9-mediated engineering of human intestinal organoids. Nat. Med..

[263] Liu X., Su Q., Zhang X., Yang W., Ning J., Jia K., Xin J., Li H., Yu L., Liao Y. (2022). Recent Advances of Organ-on-a-Chip in Cancer Modeling Research. Biosensors.

[264] Guinney J., Dienstmann R., Wang X., De Reyniès A., Schlicker A., Soneson C., Marisa L., Roepman P., Nyamundanda G., Angelino P. (2015). The consensus molecular subtypes of colorectal cancer. Nat. Med..

[265] Borelli B., Fontana E., Giordano M., Antoniotti C., Lonardi S., Bergamo F., Pietrantonio F., Morano F., Tamburini E., Boccaccino A. (2021). Prognostic and predictive impact of consensus molecular subtypes and CRCAssigner classifications in metastatic colorectal cancer: A translational analysis of the TRIBE2 study. ESMO Open.

[266] Stillman N.R., Kovacevic M., Balaz I., Hauert S. (2020). In silico modelling of cancer nanomedicine, across scales and transport barriers. npj Comput. Mater..

[267] Jean-Quartier C., Jeanquartier F., Jurisica I., Holzinger A. (2018). In silico cancer research towards 3R. BMC Cancer.

[268] Cruz S., Gomes S.E., Borralho P.M., Rodrigues C.M.P., Gaudêncio S.P., Pereira F. (2018). In silico *HCT*116 human colon cancer cell-based models en route to the discovery of lead-like anticancer drugs. Biomolecules.

[269] Gold A., Choueiry F., Jin N., Mo X., Zhu J. (2022). The Application of Metabolomics in Recent Colorectal Cancer Studies: A State-of-the-Art Review. Cancers.

[270] Gulfidan G., Turanli B., Beklen H., Sinha R., Arga K.Y. (2020). Pan-cancer mapping of differential protein-protein interactions. Sci. Rep..

[271] Uhlen M., Zhang C., Lee S., Sjöstedt E., Fagerberg L., Bidkhori G., Benfeitas R., Arif M., Liu Z., Edfors F. (2017). A pathology atlas of the human cancer transcriptome. Science.

[272] Subramanian A., Narayan R., Corsello S.M., Peck D.D., Natoli T.E., Lu X., Gould J., Davis J.F., Tubelli A.A., Asiedu J.K. (2017). A Next Generation Connectivity Map: L1000 Platform and the First 1,000,000 Profiles. Cell.

[273] Rintala T.J., Ghosh A., Fortino V. (2022). Network approaches for modeling the effect of drugs and diseases. Brief Bioinform..

