# Supplementary material for: Experimental Murine Models for Colorectal Cancer Research
Source: Cancers (Basel). 2023 Apr 30;15(9):2570. doi: 10.3390/cancers15092570 (PMC10177088; doi:10.3390/cancers15092570)
Supplement: Supplementary file 1 [file cancers-15-02570-s001.zip › cancers-2343959-supplementary.pdf]

cancers MDPI

Table S1. Colorectal cancer etiology.

Information	Details	Ref.
Screening method for CRC (≥ 45 years)	Fecal Immunochemical Test (FIT)	[3,4]
	Guaiac-based fecal occult blood test (g-FOBT)	
	Colonoscopic imaging	
Diagnostic workup for positive FIT or g-FOBT	Colonoscopy to examine the entire colon	[3,4]
Treatment for polyps, cancer, or advanced neoplasia	Surgical resection or ablation, chemo-, radio-, and immunotherapy depending on cancer stage	[5-9]
Environmental factors and CRC	90-95% of CRC cases linked to environmental factors; Linked to signal transduction cascades important for angiogenesis, apoptosis, and cell proliferation	[10-14]
Genetic or sporadic causes of CRC	80% of CRC patients have genetic or sporadic cause Hereditary types: familial adenomatous polyposis (FAP) and hereditary nonpolyposis colorectal cancer (HNPCC)	[15,16]
	Sporadic variety connected to inflammatory conditions like Crohn's disease (CD) and ulcerative colitis (UC)	
Colitis-associated CRC (CACC)	Develops in the context of chronic inflammation	[17-20]
	Patients with ulcerative colitis (UC) and Crohn's disease (CD) have 2-3x higher risk to acquire CRC	
	CACC has greater propensity for malignancy than sporadic CRC (sCRC)	
	Advanced state of CACC at diagnosis decreases life expectancy, with CRC estimated to account for 10-15% of IBD* related fatalities	
Treatment challenge for CRC	Development of irreversible metastasis and drug resistance	[21,22]
Overall survival of stage III and IV CRC	Stage III CRC has a reported incidence of 28% to 73% over the course of five years; Stage IV CRC has a reported incidence of 5% over the course of five years	[21,22]

* IBD – inflammatory bowel disease.
